# Microwave-assisted synthesis of rhodamine derivatives

**DOI:** 10.1080/17518253.2018.1472814

**Published:** 2018-05-14

**Authors:** Fasil Abebe, Treshaun Sutton, Pierce Perkins, Khalil Makins-Dennis, Angela Winstead

**Affiliations:** Department of Chemistry, Morgan State University, Baltimore, MD, USA

**Keywords:** Microwave irradiation, rhodamine-derived imines, green chemistry

## Abstract

The microwave synthesis of 12 rhodamine-derived imines is described. The present work involves condensation of rhodamine hydrazide with various aromatic aldehydes in ethanol under microwave irradiation. The results obtained indicate that, unlike classical heating, microwave irradiation results in higher yields, shorter reaction time, mild reaction condition and simple work-up procedure. The structures of synthesized compounds were confirmed by ^1^H-NMR, ^13^C-NMR, FT-IR and high-resolution mass spectra data.

## Graphical Abstract

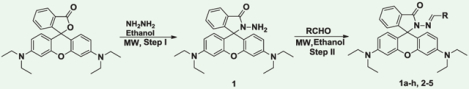

## 1. Introduction

It has been known for many years that the rhodamine compounds are widely used class of dyes due to their versatile applications in various fields (1, 2). In particular, rhodamine dyes see the widespread application as fluorescent sensors. Because of their strong fluorescence and good photostability, rhodamine derivatives have been found to have applications in laser active media (3), fluorescent markers in biology (4), imaging and bioanalysis (5), DNA sequencing (6) and fluorescence switchers and sensors (7–10).

**Scheme 1.**
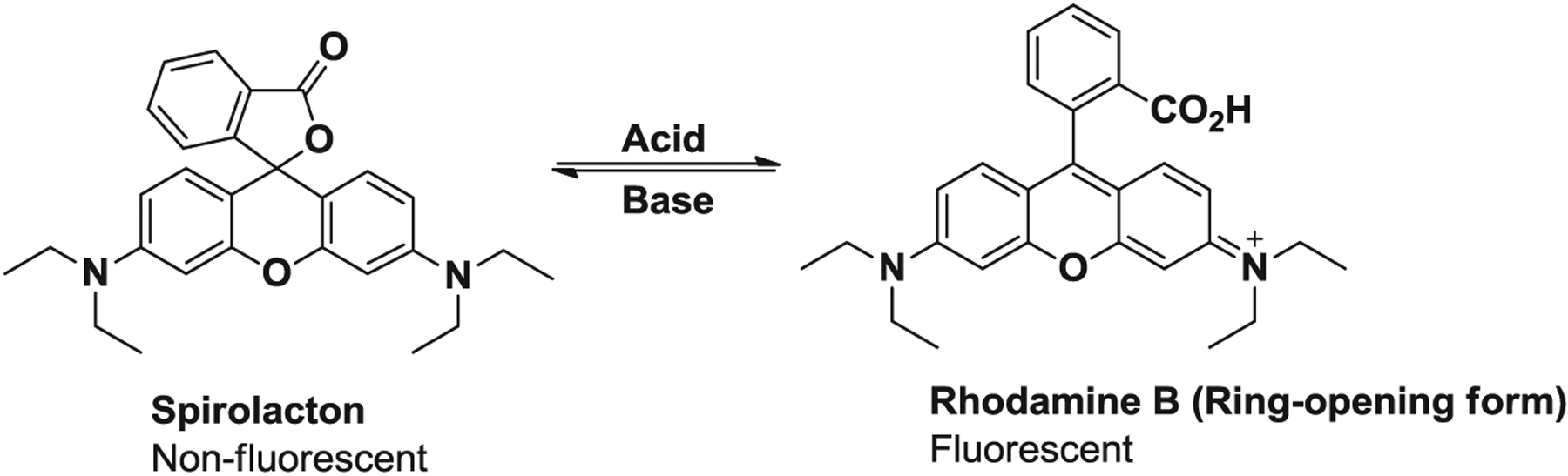
Equilibrium between cationic rhodamine B and neutral form (lacton).

**Scheme 2.**
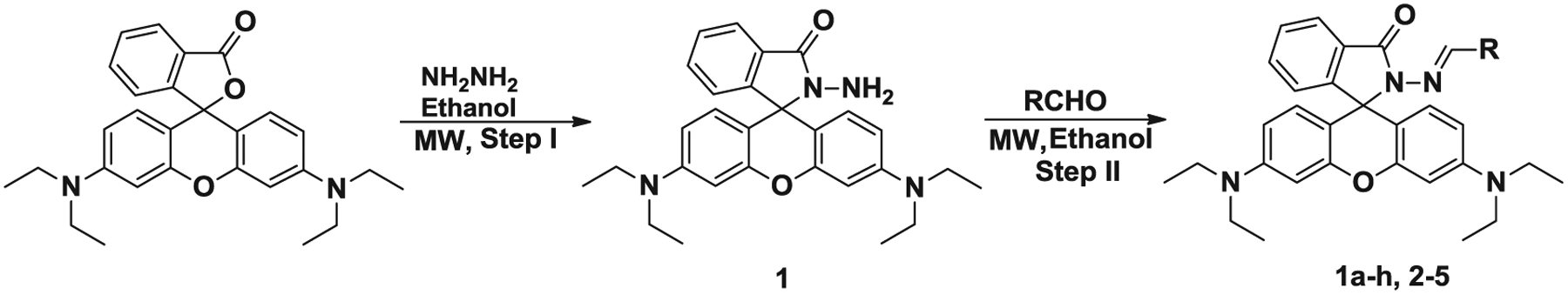
Synthetic path to prepare rhodamine-based imines (**1a–1h and 2–5).**

Rhodamine-based compounds have properties, such as very high molar extinction coefficients and high fluorescent quantum yields, ideal for use as chemical sensors (11). More recently, a strong effort has been focused on the design of ‘turn-on’ sensors, in which non-fluorescent molecules are activated in the presence of cations. The equilibrium between the non-fluorescent colorless ringclosed form (spirocyclic) and the highly fluorescent pink-colored ring-open form provides a better model for the development of turn-on sensors (Scheme 1). Cations can trigger the change in structure between the spirocyclic and open-cycle form and therefore rhodamine-based compounds have been well established as sensors for metal ions such as Cu^2+^(12, 13), Fe^3+^ (14, 15), Pb^2+^ (16, 17), Hg^2+^ (18, 19) and Cr^3+^ (20, 21). Several procedures have been developed to prepare spirocyclic rhodamines and the use of derivatives of rhodamine such as hydrazide. This highly versatile and useful compound was reported by Yang, the reaction of rhodamine B with hydrazine hydrate in methanol under reflux (22). Since then, this synthesis has been adopted as the standard method because of its convenience and versatility. A number of compounds having receptors with different coordination spheres have been prepared through the condensation of rhodamine B hydrazide with a variety of aldehydes, following the synthetic strategy present in Scheme 2. Fluorescent compounds designed to monitor and detect environmental pollutants are synthesized using reaction conditions and reagents that are not environmentally friendly. Fluorescent Schiff bases (imine) compounds have been previously synthesized using traditional organic synthetic methods, mainly via refluxing the reagents with large quantities of solvents such as chloroform, 1,2-dichloroethane, methanol and tetrahydrofuran. The researcher has true load to investigate the environmentally benign methods and reagents to prepare the chemicals because conventional methods have several drawbacks and this is overcome in the microwave-assisted synthetic method.

Microwave irradiation technique has been used for the rapid synthesis of a variety of compounds, and microwave–accelerated minimum or solvent-free organic reactions have received special attention in recent years (23). Moreover, it is considered environmentally friendly and typically offers high yields, together with simplified processing and handling compared to conventional synthesis methods. In this method, reactions occur more rapidly, safely, and with higher chemical yields, often far better than conventional methods, which require longer reaction times and larger quantities of solvents and reagents, cause environmental pollution, and contribute to health hazards (24). Microwave-assisted organic synthesis (MAOS) possesses several green attributes such as increased energy efficiencies, clean, and rapid reactions with less purification process and use of green solvents such as ethanol and water. More efficient ways of synthesizing fluorescent compounds are needed so that a variety of compounds with various spectral and chemical properties can be made available to thoroughly investigate their potential use as chemical sensors. In this work, we present an efficient, clean and straightforward procedure to prepare a series of rhodamine-derived imine compounds by MAOS under minimum solvent conditions, with short reaction times, and using ethanol as a green solvent to obtain the rhodamine derivatives from crude reaction mixtures without the use of complicated work-up. To the best of our knowledge, no examples have been reported exploring the synthesis of rhodamine imine products in a very friendly solvent-ethanol and microwave as an energy source. The influence of microwave heating on the reaction time and reaction yield was evaluated and compared to the conventional heating conditions.

## 2. Results and discussions

A Novel green method using ethanol as a solvent for the synthesis of rhodamine-based imines has been established. A total of 12 rhodamine-derived imine derivatives were synthesized using a single-mode Biotage Initiator 2.0 microwave system. The synthetic strategies adopted to yield the intermediate rhodamine hydrazide **1** and target compounds (**1a–**1**h**, **2–5**) are illustrated in Scheme 2. Rhodamine B hydrazide was synthesized from the parent rhodamine B and hydrazine in the one-step process described above using microwave irradiation and conventional oil-bath heating, by adopting the procedure reported in the literature (22). As seen in Table 1, the microwave irradiation procedure produced the rhodamine hydrazide with shorter reaction times, higher yield and easy work-up procedure. The reaction conditions for the microwave-assisted synthesis of intermediate hydrazine **1** and imines of **1a–**1**h**, **2–5** are summarized in Tables 1 and 3, respectively. The optimized conditions for each rhodamine imines were determined by conducting extensive temperature and time studies. In order to explore a simplified, less time-consuming and high yielding procedure, we have considered the use of controlled microwave heating reactor under closed-vessel conditions to optimize the reaction conditions. The reactor is equipped with a magnetic stirrer as well as temperature and power controls.

**Scheme 3.**
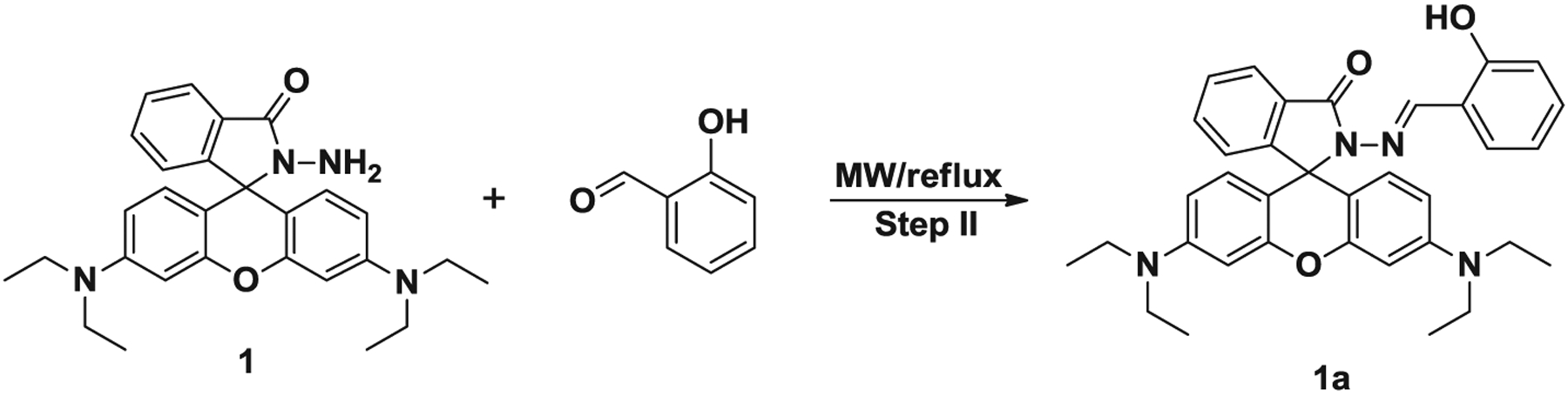
Synthetic path to prepare rhodamine-derived imine **1a.**

**Scheme 4.**
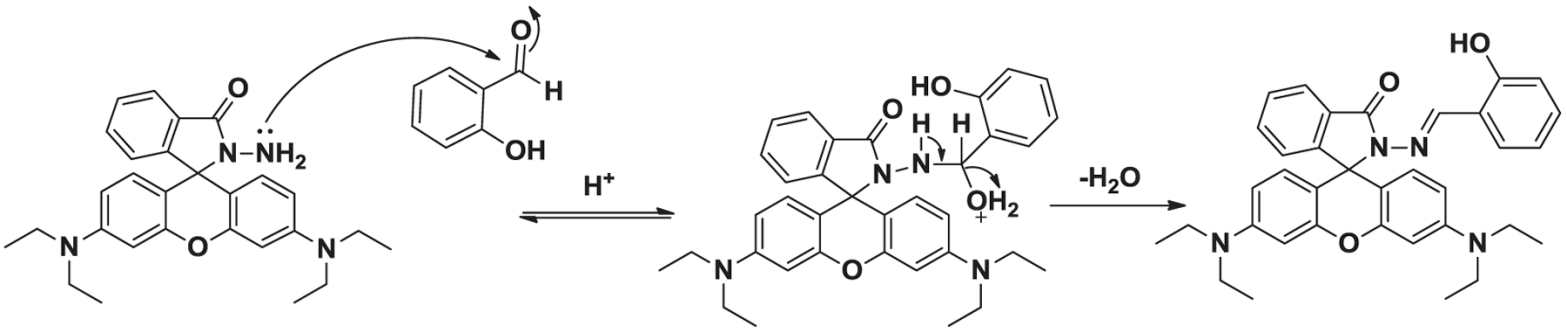
The propose mechanism of imine **1a** formation.

Initial studies were carried out with rhodamine hydrazide **1** and salicylaldehyde for imine **1a** preparation is given in Scheme 3. The mechanism is explained as shown in Scheme 4. The temperature studies range from 60 to 100°C with an initial reaction time of 10 min. The initial temperature, 60°C, was selected based upon previous studies conducted on amidation of rhodamine B (25). Upon the completion of temperature studies, time studies were conducted which ranged from 10 to 30 min. It was observed that the reaction in microwave irradiation with temperature 100°C afforded the best result with 88% product yield during 30 minutes (Table 2, entry 3). The yield was reduced to 79% by decreasing the reaction time to 10 min with the same reaction temperature. Based on the time studies, reaction yields steadily decreased from 30 to 10min. By reducing the reaction temperature to 80°C with a 10 min reaction time, resulted the product in 80% yield. These results reveal that the microwave irradiation protocol significantly accelerates the synthesis of rhodamine-based imine products, resulting in successful completion of the reaction products in only 30 min, to obtain an overall yield ranging from 57% to 93%, with the use of minimum amount of solvent. The reactions were carried out in the presence of ethanol which is classified as class III solvent having the lowest toxic potential by the Center for Drug Evaluation and Research of the USA food and drug administration.

In addition, to study the effect of solvent on the rate of reaction, we carried out the reaction with various solvents (Table 2, entries 7–10); these were selected based on their health, safety and environmental criteria (26). Due to our interest in green chemistry, minimum solvent conditions were mainly exploited. The best result was obtained with ethanol, 88% (Table 2, entry 3), methanol slightly decreased the yield, 84% (entry 7), and aqueous ethanol (1:1) yielded the product in 71% (entry 10). In the microwave-assisted condensation reaction in ethanol, the products required no rigorous purification and pure solid products were isolated from the reaction mixture. Using ethanol as a solvent, the reactions of various substituted aromatic aldehydes and rhodamine hydrazide were carried out to get imines 1a–1h, **2–5** in high yields (Table 3). It should be pointed out that no catalyst or water trapping was necessary, and purification was accomplished by simple filtration and washing the isolated product.

The microwave irradiation method was compared (in terms of time and yields %) with the conventional method for the synthesis of rhodamine-based imines as illustrated in Tables 3 and 4. The microwave method required shorter reaction times ((10–30 minutes) for completion of reaction as compared to 6–24 h by conventional method) with improved yields (57–93%) compared to the reported conventional methods (48–73%). The work-up was easy and the products were obtained in excellent to moderate yields as shown in Table 3 and 4. The results clearly demonstrate the synthesis efficiency was significantly different, as evidenced by the data of Table 3 and 4. Most significant are the substantially decreased reaction time, mild reaction conditions, no side reactions and simplicity of the reaction procedure.

The structure of the imine products **1a**–1**h**, **2**–**5** was characterized by IR, ^1^H-NMR, ^13^C-NMR and high-resolution mass spectrometry. The products were supported by the absence of the carbonyl and primary amine bands of the reactants in their IR spectra, and the presence of an imine (−C=N) band within 1612–1630 cm^−1^ region. In the ^1^H-NMR spectra, the imine protons appear within *δ* = 8.00–9.50 ppm. A broad singlet at *δ* = 10.00–13.50 was also observed for hydrogen-bonded −OH group in some imine products (1a–1h and 3) due to intramolecular H-bonding. The ^1^H-NMR spectra of imines show the typical aromatic group of signals of the xanthene scaffold, which are *δ* 6.23–6.55. In the ^13^C-NMR spectra, the imine carbon appears within 140.00–160.00 ppm and a signal at 66.0 ppm assigned to a spiro carbon atom. This value at 66.0 ppm provides evidence of the presence of the rhodamine in the spiro-lactam form and consistent with the values described in the literature for these compounds (32).

The protocol for the synthesis of imines under microwave irradiation in the absence of any catalyst or solid support affords a clean, efficient and environmentally friendly method. Therefore, this method offers an easy practical access for the production of a series of rhodamine-based imine products. There is a high probability that microwave heating also could be applied to make similar systems such as fluorescein and other xanthene-based dyes. This would be highly advantageous for imaging, chemical sensor and drug discovery laboratories where small amounts of different analogues have to be synthesized in short periods of time, as well as combinatorial synthesis of new libraries of compounds (33). With the decrease in the use of solvents, it was possible to obtain more consistent products in line with the principles of green chemistry. The spectroscopic data were in good agreement with the proposed structures of the prepared rhodamine derivatives. Rhodamine derivatives **1a–1h** and **2–5** were characterized by ^1^H-NMR, ^13^C-NMR and high-resolution mass spectroscopy (Experimental Section).

## 3. Experimental section

### 3.1. Materials and apparatus

All microwave reactions were conducted using the single-mode Biotage Initiator 2.0 (Biotage, Uppsala, Sweden); all the products were characterized by spectral data (HR-ESI-MS, ^1^H-NMR, ^13^C-NMR, FT-IR). ^1^H- and ^13^C-NMR spectra (400 MHz for proton and 100 MHz for carbon) were obtained on a Bruker-Vance 400 MHz spectrometer (Bruker, Rheinstetten, Germany), using CDCl_3_ and DMSO-d_6_ as solvents. Tetramethylsilane was used as an internal standard. Data for ^1^H-NMR are reported as follows: chemical shifts (*δ* ppm), multiplicity (s, singlet; d, doublet; dd, doublet of doublets; t, triplet; m, multiplet), integration and coupling constant (Hz). Chemical shifts (*δ*) and *J* values are reported in ppm and Hz, respectively, relative to the solvent peak CDCl_3_ at 7.2 ppm for protons and 77 ppm for carbon atoms. NMR Spectra were analyzed using MestReNova software (version 10, Mestrela research, Feliciano Barrera-Bajo, Spain). The IR spectrum was obtained using FT-IR spectrometer **(**Shimadzu, IRAffinity-1S, and Columbia, MD, USA). High-resolution ESI-MS was acquired with a Bruker Apex-Qe instrument. Melting points (uncorrected) were measured on a Fisher-Jones melting point apparatus. All reagents and chemicals were obtained from Aldrich Chemical Company (St. Louis, MO, USA) and Alfa Aesar (Ward Hill, MA, USA) and were used without further purification.

### 3.2. General procedure for the synthesis of rhodamine-derived imines from hydrazide

The protocol to synthesize compounds **1a–**1**h and 2–5** involves the reaction of rhodamine B with hydrazine hydrate (80%) in ethanol (step I), followed by condensation of the resulting rhodamine hydrazide **1** (100 mg, 0.219 mmol) with equimolar amounts of various aromatic aldehydes in ethanol (2 mL, step II), as described in Scheme 2. The resulting mixture was stirred to make it homogeneous and then placed in the cavity of a biotage microwave reactor. The closed reaction vessel was run under pressure and the reaction was irradiated according to the parameters described in Table 2. These reactions were performed safely at a maximum temperature of 100°C. However, reactions can safely be performed at pressures up to 20 bar and temperatures ranging from 40°C to 250°C. After cooling to room temperature, the resulting solid was filtered and washed three times with cold ethanol. After drying, the product was isolated to give in the desired yield.

### 3.3. Physical and spectroscopic data of the isolated products

#### (E)-3′,6′-bis(diethylamino)-2-(2-hydroxybenzylideneamino)spiro[isoindoline-1,9′-xanthene]-3-one (1a).

A mixture of **1** (100 mg, 0.219 mmol), salicylaldehyde (27 mg, 0.221 mmol) and ethanol (2 mL) was placed in a 10 mL reaction vial. The resulting mixture was stirred to make it homogeneous and it was placed in the cavity of a biotage microwave reactor. The closed reaction vessel was run under pressure and irradiated at a temperature of 100°C for 30 minutes. After cooling to room temperature, the resulting white solid was filtered and washed three times with cold ethanol. 88% yield; m.p. 155–160° C ^1^H-NMR (DMSO-d_6_), *δ* (ppm): 10.46 (1H, s, −OH), 9.09 (1H, s, −CH=N), 7.90 (1H, d, *J* = 6.6 Hz, Ar-H), 7.66–7.51 (2H, m, Ar-H), 7.30 (1H, d, *J* = 8.7 Hz, Ar-H), 7.21 (1H, dd, -Ar), 7.12 (1 H, d, *J* =8.6, -Ar), 6.83 (H, d, -Ar), 6.47 (2H, d, *J* = 8.8 Hz, Ar-H), 6.42 (2H, s, Ar-H), 6.34 (1H, d, Ar-H), 6.33 (1H, d, Ar-H), 3.32–3.25 (8H, m, NCH_2_CH_3_), 1.0.7 (12H, t, *J* = 7.0 Hz, NCH_2_CH_3_). ^13^C-NMR (DMSO-d_6_), *δ* (ppm): 164.1, 157.5, 153.3, 151.5, 150.5, 149.1 (−CH=N), 134.6, 132.2, 129.9, 129.5, 129.2, 128.1, 124.4, 123.6, 120.0, 119.3, 116.9, 108.7, 105.3, 97.8, 66.1(spiro carbon), 44.2 (NCH_2-_CH_3_), 12.9 (NCH_2_CH_3_). HRMS (ESI): m/z Calcd for C_35_H_36_N_4-_O_3_: 561.2860; Found: 561.2863 [M+H]^+^.

#### (E)-3′ ,6′-bis(diethylamino)-2-(4-hydroxybenzylideneamino)spiro[isoindoline-1,9′-xanthene]-3-one (1b).

White solid; m.p.153–155°C; ^1^H-NMR (DMSO-d_6_), *δ* (ppm): 9.90 (1H, s, −OH), 8.79 (1H, s, −CH=N), 7.87 (1H, d, *J* = 6.6 Hz, -Ar), 7.61–7.51 (2H, m, -Ar), 7.26 (2H, d, *J* = 8.7 Hz, -Ar), 7.07 (1H, d, *J* = 7.0 Hz, -Ar), 6.72 (2H, d, *J* = 8.6, -Ar), 6.42 (2H, d, *J* =2.4 Hz, -Ar), 6.40 (2H, d, *J* = 8.8 Hz, Ar-H), 6.32 (2H, dd, *J* =8.9 and *J* =2.4 Hz, Ar-H), 3.32–3.25 (8H, m, NCH_2_CH_3_), 1.0.7 (12H, t, *J* = 7.0 Hz, NCH_2_CH_3_). ^13^C-NMR (DMSO-d_6_), *δ* (ppm): 163.89, 160.11, 153.19, 151.63, 149.75, 148.86 (−CH=N), 134.03, 129.66, 129.15, 128.17, 126.05, 124.24, 123.33, 116.17, 108.42, 106.19, 97.77, 65.85 (spiro carbon), 55.45, 44.16 (NCH_2_CH_3_), 12.94 (NCH_2_CH_3_). HRMS (ESI): m/z Calcd for C_35_H_36_N_4_O_3_: 561.3860; Found: 561.3863 [M+H]^+^.

#### (E)-3′,6′-bis(diethylamino)-2-(2-hydroxy-5-nitrobenzylide-neamino)spiro[isoindoline-1,9′-xanthene]-3-one (1c).

White solid; m.p. 232–234°C; ^1^H-NMR (CDCl_3_), *δ* (ppm):12.05 (1H, s, −OH), 8.92 (1H, s, −CH=N), 8.08–8.0 (3H, m, -Ar), 7.55 (2H, m, -Ar), 7.19 (1H, d, *J* = 6.6 Hz, -Ar), 6.96–6.50 (4H, m, -Ar), 6.23 (2H, d, *J* =7.5 Hz, -Ar), 3.34 (8H, q, NCH_2_CH_3_), 1.26 (12H, t, *J* = 6.6 Hz, NCH_2_CH_3_). ^13^C-NMR (CDCl_3_), *δ* (ppm): 164.0, 165.0, 152.7, 151.0, 150.0, 148.5, 140.0 (−CH=N), 135.5, 130.1, 128.9, 128.5, 127.5, 124.1, 123.8, 119.3, 118.0, 108.1, 105.5, 97.5, 80.9, 66.5 (spiro carbon), 44.6 (NCH_2_CH_3_), 12.4 (NCH_2_CH_3_). HRMS (ESI): m/z Calcd for C_35_H_35_N_5_O_5_: 605.2071; Found: 605.2076 [M+H]^+^.

#### (E)-3′,6′-bis (diethylamino)-2-(2-hydroxy-3-methoxybenzylideneamino) spiro [isoindoline-1, 9′-xanthene]-3-one (1d).

Pink solid; m.p. 222–224°C; ^1^H-NMR (CDCl_3_), *δ* (ppm):10.7 (1H, s, −OH), 9.2 (1H, s, −CH=N), 7.86 (1H, d, *J* = 6.6 Hz, -Ar), 7.48 (2H, m, -Ar), 7.1 (1H, d, *J* = 6.6 Hz, -Ar), 6.7 (3H, m, -Ar), 6.49 (2H, d, -Ar), 6.40 (2H, s, -Ar), 6.23 (2H, d, *J* = 7.5 Hz, -Ar), 3.80 (3H, s, −OCH_3_), 3.31 (8H, q, NCH_2_CH_3_), 1.16 (12H, t, *J* = 6.6 Hz, NCH_2_CH_3_). ^13^C-NMR (CDCl_3_), *δ* (ppm): 163.6, 152.7, 148.5, 146.6 (−CH=N), 138.5, 138.1, 137.7, 134.0, 128.9, 128.5, 127.5, 123.1, 121.8, 121.3, 108.1, 108.0, 106.5, 104.8, 97.3, 80.9, 65.5 (spiro carbon), 56.1, 43.6 (NCH_2_CH_3_), 12.4 (NCH_2_CH_3_). HRMS (ESI): m/z Calcd for C_36_H_38_N_4_O_4_: 591.2966; Found: 591.2969 [M+H]^+^.

#### (E)-3′,6′-bis(diethylamino)-2-(4-diethylamino)-2-hydroxybenzylideneamino)spiro [isoindoline-1, 9′-xanthene]-3-one (7e).

Yellow solid; m.p. 214–217°C; ^1^H-NMR (CDCl_3_), *δ* (ppm):10.96 (1H, s, Phen-OH), 9.19 (1H, s, −CH=N), 7.93 (1H, d, *J* = 6.6 Hz, Ar-H), 7.46 (2H, dd, Ar-H), 7.13 (1H, d, *J* = 6.6 Hz, Ar-H), 6.90 (1H, d, Ar), 6.49–6.47 (4H, m, Ar-H), 6.23 (2H, dd, *J* = 7.5 Hz, -Ar), 6.09 (2H, m, Ar-H), 3.27 (12H, m, NCH_2_CH_3_), 1.11 (18H, m, NCH_2_CH_3_). ^13^C-NMR (CDCl_3_), *δ* (ppm): 163.7, 160.7, 154.8, 153.7, 150.6, 149.0 (−CH=N), 133.5, 132.9, 130.0, 128.9, 128.2, 124,123.0, 108.1, 107.6, 106.0, 103.4, 98.1, 66.5 (spiro carbon), 44.6, (NCH_2_CH_3_), 44.4 (NCH_2_CH_3_), 12.8 (NCH_2_CH_3_), 12.7 (NCH_2_CH_3_). HRMS (ESI): m/z Calcd for C_39_H_45_N_5_O_3_: 632.3595; Found: 632.3597 [M+H]^+^.

#### (E)-2-(2,6-dichlorobenzylideneamino)-3′,6′-bis(diethylamino)spiro [isoindoline-1, 9′-xanthene]-3-one (1f).

White solid; m.p. 172–175°C; ^1^H-NMR (CDCl_3_), *δ* (ppm): 8.57 (1H, s, −CH=N), 8.02 (1H, d, *J* = 6.6 Hz, -Ar), 7.52–7.47 (2H, m, -Ar), 7.25 (3H, m, -Ar), 7.10 (1H, q, -Ar), 6.58 (2H, d, *J* = 7.5 Hz, -Ar), 6.40 (2H, s, -Ar), 6.32 (2H, dd, *J* = 8 Hz and *J* = 2.2 Hz, Ar), 3.35 (8H, q, NCH_2_CH_3_), 1.18 (12H, t, *J* = 6.6 Hz, NCH_2_CH_3_). ^13^C-NMR (CDCl_3_), *δ* (ppm): 163.6, 152.7, 148.5, 146.6 (−CH=N), 138.5, 138.1, 137.7, 134.0, 128.9, 128.5, 127.5, 123.1, 121.8, 121.3, 108.1, 108.0, 106.5, 104.8, 97.3, 80.9, 65.5 (spiro carbon), 56.1, 43.6 (NCH_2_CH_3_), 12.4 (NCH_2_CH_3_). HRMS (ESI): m/z Calcd for C_35_H_34_Cl_2_N_4-_O_2_: 613.2131; Found: 613.2133[M+H]^+^.

#### (E)-3′,6′-bis(diethylamino)-2-(2-hydroxy-3,5-dinitrobenzylideneamino)spiro[isoindoline-1,9′-xanthene]-3-one (1g).

Yellow solid; m.p. 237–239°C; ^1^H-NMR (CDCl_3_), *δ* (ppm):13.11 (1H, s, −OH), 8.54 (1H, s, −CH=N), 8.5 (1H, s, -Ar), 8.11 (1H, s, -Ar), 8.00 (1H, d, *J* = 6.6 Hz, -Ar), 7.50 (2H, m, -Ar), 7.10 (1H, d, *J* = 6 Hz, -Ar), 6.50–6.43 (4H, m, -Ar), 6.30 (2H, s, -Ar), 3.28 (8H, q, NCH_2_CH_3_), 1.12 (12H, t, *J* = 6.6 Hz, NCH_2_CH_3_) ^13^C-NMR (CDCl_3_), *δ* (ppm): 162.1, 150.7, 148.5, 145.6 (−CH=N),137.5, 136.1, 136.7, 134.0, 128.9, 128.5, 127.5, 123.1, 120.8, 120.3, 107.0, 108.0, 106.5, 103.8, 97.3, 80.9, 65.0 (spiro carbon), 56.1, 43.6 (NCH_2_CH_3_), 12.4 (NCH_2_CH_3_). HRMS (ESI): m/z Calcd for C_35_H_34_N_6_O_7_: 651.2561; Found: 651.2564 [M+H]^+^.

#### (E)-2-(5-bromo-2-hydroxy-3-methoxybenzylideneamino)-3′,6′-bis (diethylamino)spiro[isoindoline-1,9′-xanthene]-3-one (1h).

Brown solid; m.p. 228–232°C; ^1^H-NMR (CDCl_3_), *δ* (ppm): 11.11 (1H, s, −OH), 8.94 (1H, s, −CH=N), 7.96 (1H, t, *J* = 6.6 Hz, -Ar), 7.49 (2H, m, -Ar), 6.86 (1H, d, *J* = 6.6 Hz, -Ar), 7.50 (2H, s, -Ar), 6.51–6.43 (4H, m, -Ar), 6.25 (2H, d, *J* =7.5 Hz, -Ar), 3.82 (3H, s, −OCH_3_), 3.31 (8H, q, NCH_2_CH_3_), 1.16 (12H, t, *J* = 6.6 Hz, NCH_2_CH_3_) ^13^C-NMR (CDCl_3_), *δ* (ppm): 163.6, 152.7, 148.5, 146.6 (−CH=N), 138.5, 138.1, 137.7, 134.0, 128.9, 128.5, 127.5, 123.1, 121.8, 121.3, 108.1, 108.0, 106.5, 104.8, 97.3, 80.9, 65.5 (spiro carbon), 56.1, 43.6 (NCH_2_CH_3_), 12.4 (NCH_2_CH_3_). HRMS (ESI): m/z Calcd for C_36_H_37_BrN_4_O_4_: 669.2071; Found: 669.2076 [M+H]^+^.

#### (E)-3′,6′-bis(diethylamino)-2-[(5-methylthiophene-2-yl)methylamino-4a′,9a′-dihydrospiro[isoindoline-1,9′-xanthene]-3-one (2).

Pale yellow solid; m.p.178–182°C; ^1^H-NMR (DMSO-d_6_), *δ* (ppm): 9.01 (1H, s, −CH=N), 7.87 (1H, d, *J* = 6.6 Hz, -Ar), 7.61–7.51 (2H, m, -Ar), 7.07 (2H, d, *J* = 6.6 Hz, -Ar), 6.72 (1H, d, *J* = 6.6 Hz, -Ar), 6.45–6.31 (4H, m, -Ar), 6.3 (2H, m, Ar-H), 3.23 (8H, q, NCH_2_CH_3_), 2.4 (3H, s, −CH_3_), 1.09 (12H, t, *J* = 6.6 Hz, NCH_2_CH_3_). ^13^C-NMR (CDCl_3_), *δ* (ppm): 165.0, 153.0, 148.5, 146.6 (−CH=N), 138.5, 138.1, 137.7, 134.0, 128.9, 128.5, 127.5, 123.1, 121.8, 121.3, 108.1, 108.0, 106.5, 104.8, 97.3, 80.9, 64.0 (spiro carbon), 56.1, 43.6 (NCH_2_CH_3_), 12.4 (NCH_2_CH_3_). HRMS (ESI): m/z Calcd for C_34_H_36_N_4_O_2_S: 565.2632; Found: 565.2634 [M+H]^+^.

#### (E)-3′,6′-bis(diethylamino)-2-[(2-hydroxynaphthalen-1-yl)methyleneamino)spiro [isoindoline-1,9′-xanthene]-3-one (3).

Yellow solid; m.p. 206–208°C; ^1^H-NMR (CDCl_3_), *δ* (ppm): 12.22 (1H, s, −OH), 9.85 (1H, s, −CH=N), 7.84 (1H, t, *J* = 6.6 Hz, -Ar), 7.64 (1H, m, -Ar), 7.53 (2H, m, -Ar), 7.30 (2H, d, -Ar), 7.51 (2H, m, -Ar), 7.20–7.07 (2H, m, -Ar), 6.55–6.51 (4H, m, -Ar), 6.27–6.24 (2H, dd, *J* = 6.6 Hz, -Ar), 3.29 (8H, q, NCH_2_CH_3_), 1.11 (12H, t, *J* = 6.6 Hz, NCH_2_CH_3_). 164.6, 157.8, 153.4, 151.7, 148.8, 147.6 (N = C–H), 137.6, 133.1, 131.9, 130.3, 129.2, 128.1, 127.0, 126.7, 124.0, 123.2, 116.8, 112.9, 108.1, 107.9, 106.5, 104.6, 79.9, 66.3 (spiro carbon), 56.7, 44.3 (NCH_2_CH_3_), 12.7(NCH_2_CH_3_); HRMS (ESI): m/z Calcd for C_39_H_38_N_4_O_3_: 611.3017; Found: 611.3021 [M+H]^+^.

#### (E)-3′,6′-bis(Diethylamine)-2-[(2-methoxynaphthalene-1-yl)methyleneamino]spiro[isoindoline-1,9′-xanthen]-3-one(4).

White solid; m.p. 244–246°C; ^1^H-NMR (CDCl_3_), *δ* (ppm): 9.63 (1H, s, N = C–H); 8.77 (1H, d, *J* =7.4 Hz, H-Ar), 7.74 (1H, d, *J* = 8.4 Hz, H-Ar), 7.71 (1H, d, *J* = 8.0, H-Ar), 7.63 (1H, d, 7.7 Hz, H-Ar), 7.51–7.48 (2H, m, H-Ar), 7.27–7.15 (2H, m, H-Ar), 7.12 (1H, d, *J* = 8.4 Hz), 7.09 (1H, d, *J* = 4.9 Hz), 6.63 (2H, d, 8.8 Hz), 6.44 (2H, d, 2.2 Hz), 6.28 (2H, dd, *J* = 8.8 Hz, 2.6 Hz), 3.82 (3H, s, OCH_3_), 3.31 (8H, q, *J* = 6.9 Hz, NCH_2_CH_3_), 1.14 (12H, t, *J* = 6.9 Hz, NCH_2_CH_3_). ^13^C-NMR (CDCl_3_), *δ* (ppm): 164.6, 157.8, 153.4, 151.7, 148.8, 147.6 (N = C–H), 137.6, 133.1, 131.9, 130.3, 129.2, 128.1, 127.0, 126.7, 124.0, 123.2, 116.8, 112.9, 108.1, 107.9, 106.5, 104.6, 79.9, 66.3 (spiro carbon), 56.7, 44.3 (NCH_2_CH_3_), 12.7(NCH_2_CH_3_); HRMS (ESI): m/z Calcd for C_40_H_40_N_4_O_3_: 625.3173; Found: 625.3176 [M+H]^+^.

#### (E)-3′,6′-bis(diethylamino)-2-[(6-methyl-4-oxo-4H-chromen-3-yl)methylenamino)-4a′,9a′-dihydrospiro[isoindoline-1,9′-xanthene]-3-one(5).

White solid; m.p. 245–247°C; ^1^H-NMR (CDCl_3_), *δ* (ppm): 8.69 (1H, s, −CH=N), 8.33 (1H, s, -Ar), 7.85 (1H, d, *J* = 6.6 Hz, -Ar), 7.82 (1H, s, -Ar), 7.40 (3H, m, -Ar), 7.23 (1H, d, -Ar), 7.19 (1H, d, -Ar), 6.44–6.39 (4H, m, -Ar), 6.18 (2H, d, *J* = 7.5 Hz, -Ar), 3.24 (8H, q, NCH_2_CH_3_), 2.32 (3H, s, −OCH_3_), 1.07 (12H, t, *J* = 6.6 Hz, NCH_2_CH_3_). ^13^C-NMR (CDCl_3_), *δ* (ppm): 175.6, 165.0, 154.4, 153.8, 153.2, 152.0, 148.9, 140.4 (−CH=N), 135.4, 134.8, 133.4, 128.9, 128.2, 127.9, 125.3, 123.8, 123.4, 119.7, 117.9, 107.9, 105.7, 98.1, 66.0 (spiro carbon), 44.3 (NCH_2_CH_3_), 20. 9, 12.6 (NCH_2_CH_3_). HRMS (ESI): m/z Calcd for C_39_H_38_N_4_O_4_: 627.2966; Found: 627.2969 [M+H]^+^.

## 4. Conclusions

The method shown here is the most convenient way to synthesize the rhodamine-derived imines, in which microwave irradiation plays an important role for promoting the condensation reaction of aromatic aldehyde and rhodamine hydrazide. In all the reactions reported in this paper, we proceeded under minimum solvent conditions, wherein the reactions are completed within a few minutes and in good yields (30–60%, higher than those of conventional methods) under microwave irradiation. Thus, we conclude that single-mode microwave system has provided substantially decreased reaction times, simplicity of reaction procedure and increased yields observed for reactions conducted.

## Supplementary Material

Supplement

## Figures and Tables

**Table 1.** Optimization of microwave (MW) assisted method for the preparation of hydrazide **1**.

Entry	Method	Time (min)	Temp. (°C)	Yield (%)
1	MW in ethanol	5	80	86
2	MW in ethanol	10	80	82
3	MW without ethanol	15	80	75
4	MW in ethanol	5	100	85
5	Oil-bath in ethanol	180	80	73

**Table 2.** Optimization of microwave (MW) assisted method for the preparation of imine **1a**.

Entry	Method	Time (min)	Temp. (°C)	Yield (%)
1	MW in ethanol	10	100	79
2	MW in ethanol	20	100	80
3	MW in ethanol	30	100	88
4	MW in ethanol	10	80	80
5	MW in ethanol	20	80	73
6	MW in ethanol	10	60	70
7	MW in methanol	10	80	84
8	MW in methanol	10	100	82
9	Oil-bath in ethanol	360	80	70
10	aqueous ethanol	10	80	71

**Table 3.** Synthesis of rhodamine derivatives using microwave (MW) heating method.

Entry	*R*	Product (imines)	MW Time (min)/Temp. (°C)	MW Yield^a^ (%)	Lit. time (h)	Lit. yield (%) (Ref.)	m.p. (°C)
**1**	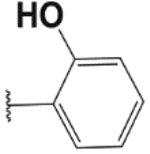	**1a**	30/100	88	6	76 (27)	158–160
**2**	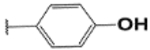	**1b**	30/100	92	6	90 (28)	153–155
**3**	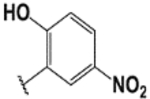	**1c**	20/100	82	24	73 (29)	232–234
**4**	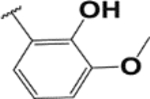	**1d**	20/80	63	N/A	N/A	222–224
**5**	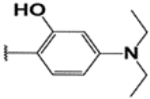	**1e**	20/100	78	6	57 (30)	214–217
**6**	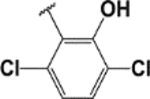	**1f**	20/100	81	N/A	N/A	172–175
**7**	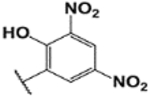	1**g**	20/100	57	N/A	N/A	237–239
**8**	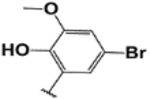	**1h**	10/80	80	N/A	N/A	228–232
**9**	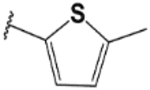	**2**	20/80	73	N/A	N/A	178–182
**10**	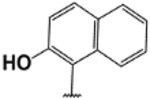	**3**	10/100	93	24	73 (31)	206–208
**11**	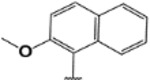	**4**	10/100	92	N/A	N/A	244–246
**12**	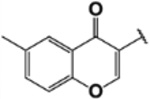	**5**	10/80	88	N/A	N/A	245–247

Note: Yields refer to pure isolated products; lit. yield (literature yield, conventional heating).

**Table 4.** Yields of rhodamine-derived imines (**1a–1h, 2–5**) under conventional heating and MW irradiation conditions.

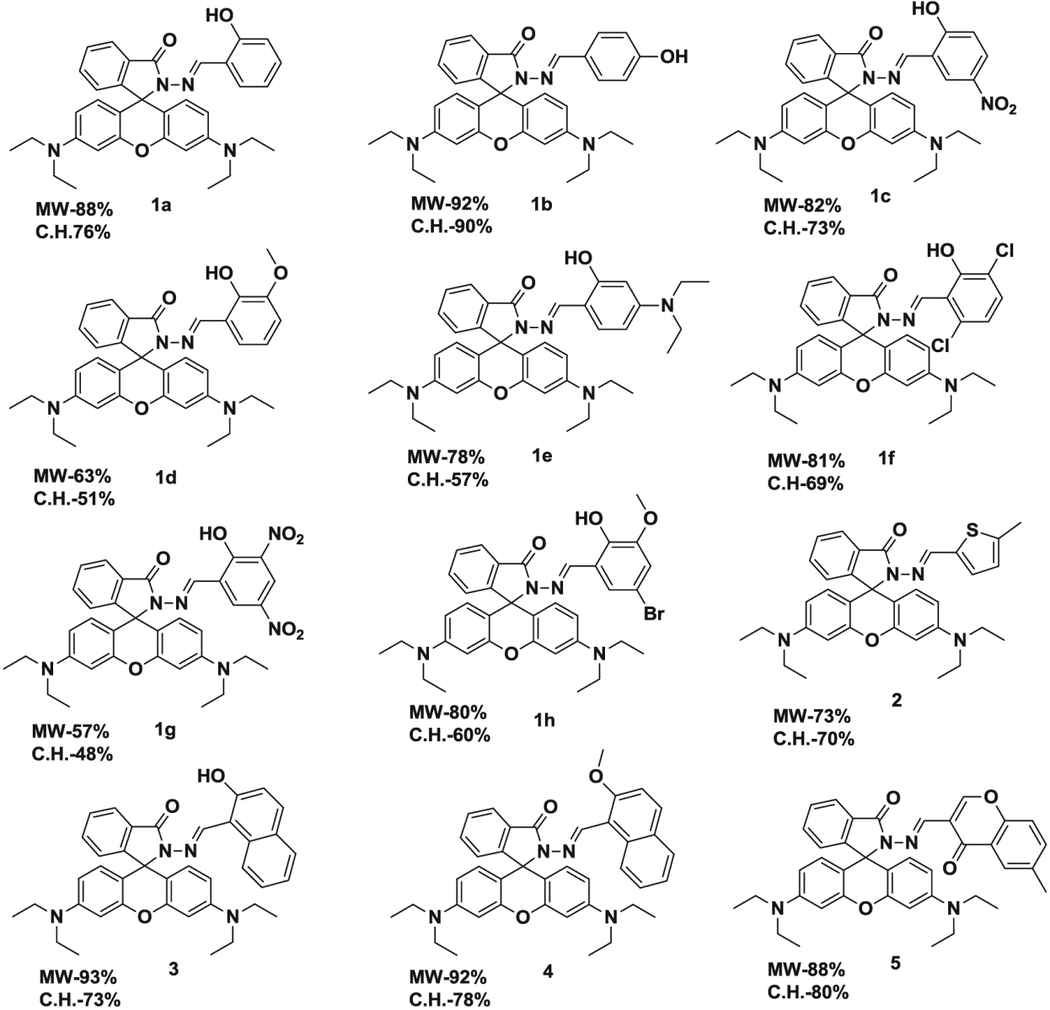

Note: C.H. – conventional heating; MW – microwave irradiation.
