# Supplementary material for: Microwave-assisted synthesis of rhodamine derivatives
Source: Green Chem Lett Rev. Author manuscript; Available in PMC 2020 Mar 19. (PMC7082095; doi:10.1080/17518253.2018.1472814)
Supplement: Supplement [file NIHMS1561965-supplement-Supplement.pdf]

Microwave-assisted synthesis of rhodamine derivatives

Fasil Abebe*, Treshaun Sutton, Pierce Perkins, Khalil Makins-Dennis and Angela Winstead

Department of Chemistry, Morgan State University, Baltimore, MD 21251, USA

*Author to whom correspondence should be addressed**;** E-mail: Fasil.Abebe@morgan.edu.

General procedure for the synthesis of compounds 1a-h and 2-5 with microwave

The protocol to synthesize compounds **1a-h and 2-5** involves the reaction of rhodamine B with hydrazine hydrate (80%) in ethanol (step I), followed by condensation of the resulting rhodamine hydrazide **1** (100mg, 0.219mmol) with equimolar amounts of various aromatic aldehydes in ethanol (2 mL, step II), as described in Scheme 2. The resulting mixture was stirred to make it homogeneous and then placed in the cavity of a biotage microwave reactor. The closed reaction vessel was run under pressure and the reaction was irradiated according to the parameters described in Table 2. These reactions were performed safely at a maximum temperature of 100 °C. However, reactions can safely be performed at pressures up to 20 bar and temperatures ranging from 40 °C to 250 °C. After cooling to room temperature, the resulting solid was filtered and washed three times with cold ethanol. After drying, the product was isolated to give in the desired yield.

2-amino-3’,6’- bis(diethylamino)spiro[isoindoline-1,9’-xanthen]-3-one (**1**)
A mixture of rhodamine B 1 (0.12 g, 0.25 mmol), an excess of hydrazine hydrate (80%) (0.3 mL) and ethanol (2 mL) was placed in a 10 mL reaction vial. The resulting mixture was stirred to make it homogenous and it was placed in the cavity of Biotage microwave reactor irradiated for 5 min at 100°C. After cooling to room temperature, the resulting solid was filtered and washed 3 times with water. After drying, **1** was isolated to give 85% yield. m.p. 176-177; ^{1}H-NMR ($CDCl_3$), δ (ppm): 1.14 (12 H, t, J=7.2 Hz), NCH_2CH_3), 3.31 (8 H, q, J=7.2 Hz, NCH_2CH_3), 3.64 (2 H, broad s, NH_2), 6.31 (2 H, dd, J=8.8 and J=2.4 Hz, H-2, 7), 6.44 (2 H, d, J=2.4 Hz), H-4, 5), 6.48 (2 H, d, J=8.8 Hz, H-1, 8), 7.12-7.14 (1 H, m, Ar-H), 7.46-7.50 (2 H, m, Ar-H), 7.95-7.97 (1 H, m, Ar-H). ^{13}C-NMR ($CDCl_3$), δ (ppm): 12.7 (NCH_2CH_3), 44.4 (NCH_2CH_3), 66.0 (spiro carbon), 77.1, 98.0, 104.6, 108.0, 108.1, 122.9, 123.8, 128.1, 130.0, 132.5, 148.9, 151.6, 153.9, 166.

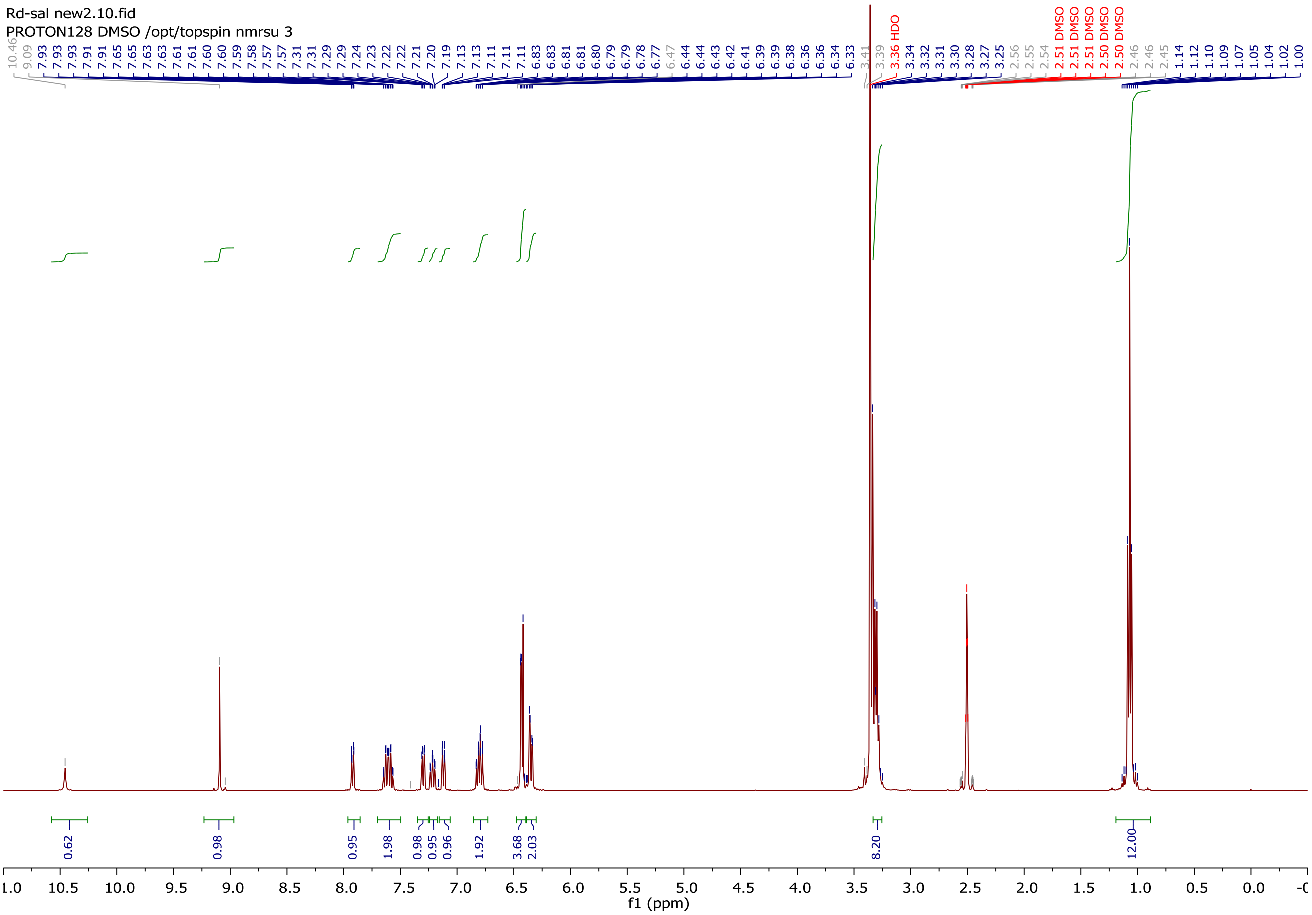

Fig S1: ^{1}H-NMR spectra for compound **1a** (DMSO, 400MHz)

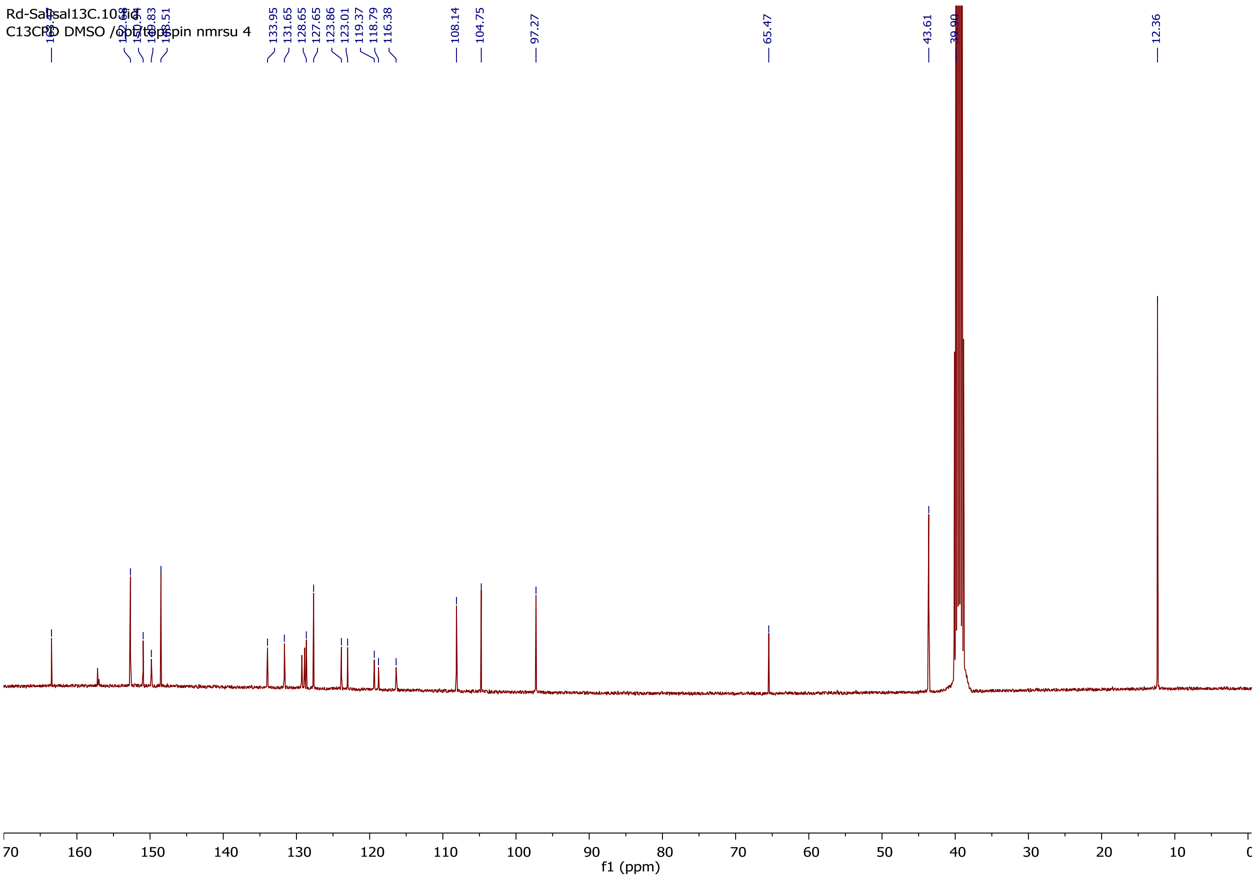

Fig S2: ^{13}C-NMR spectra for compound **1a** (DMSO, 400MHz)

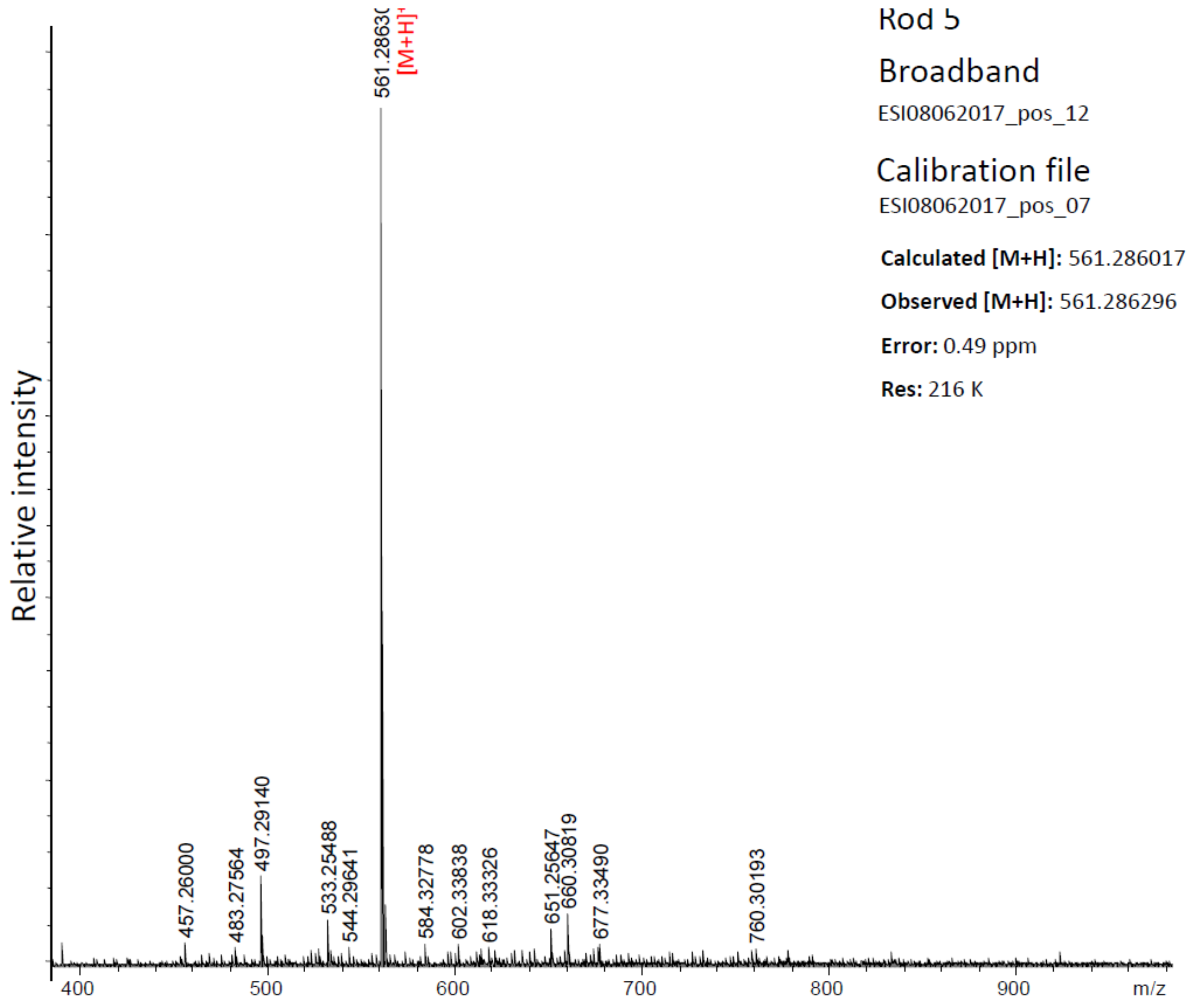

Fig. S3: HRMS (ESI) spectrum of compound **1a**

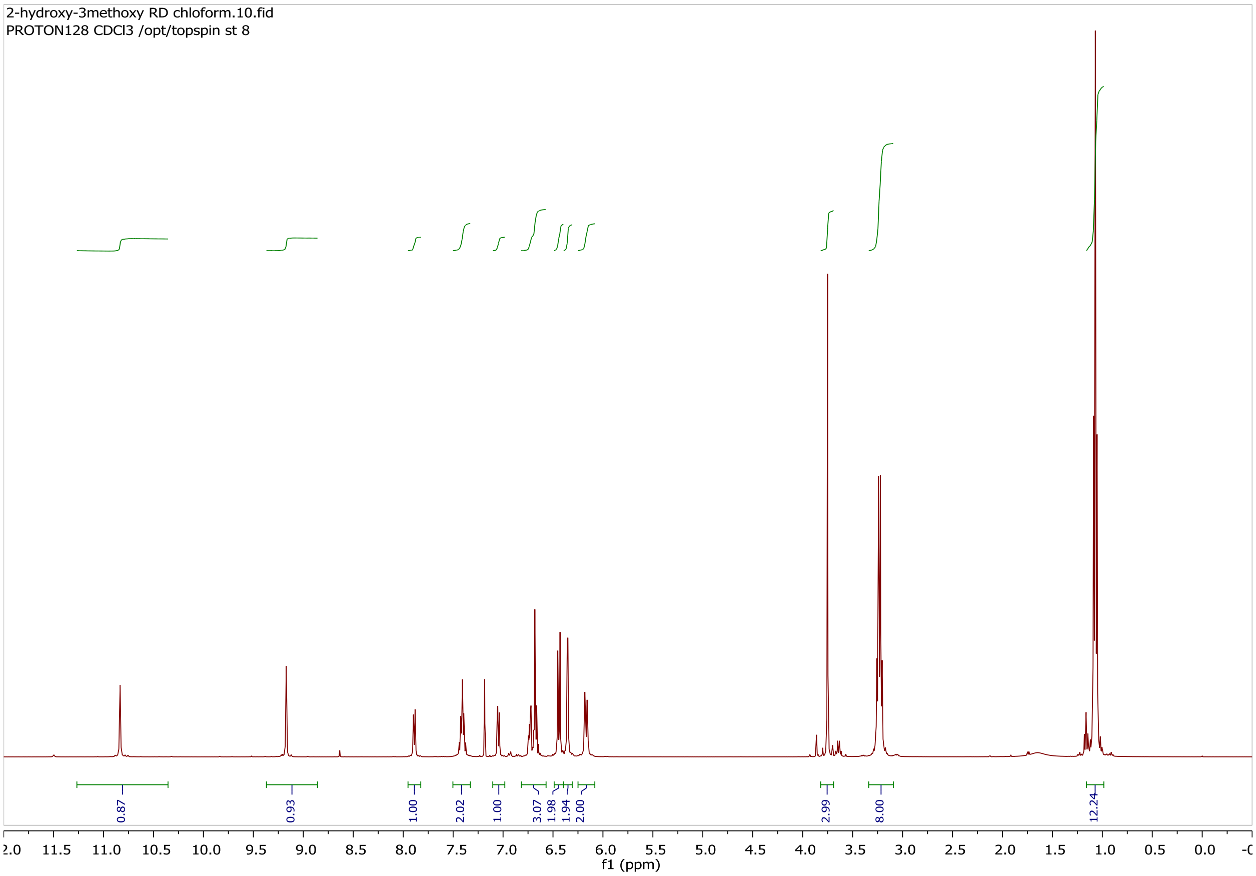

Fig S4: ^{1}H-NMR spectra for compound **1d** ($CDCl_3$, 400MHz)

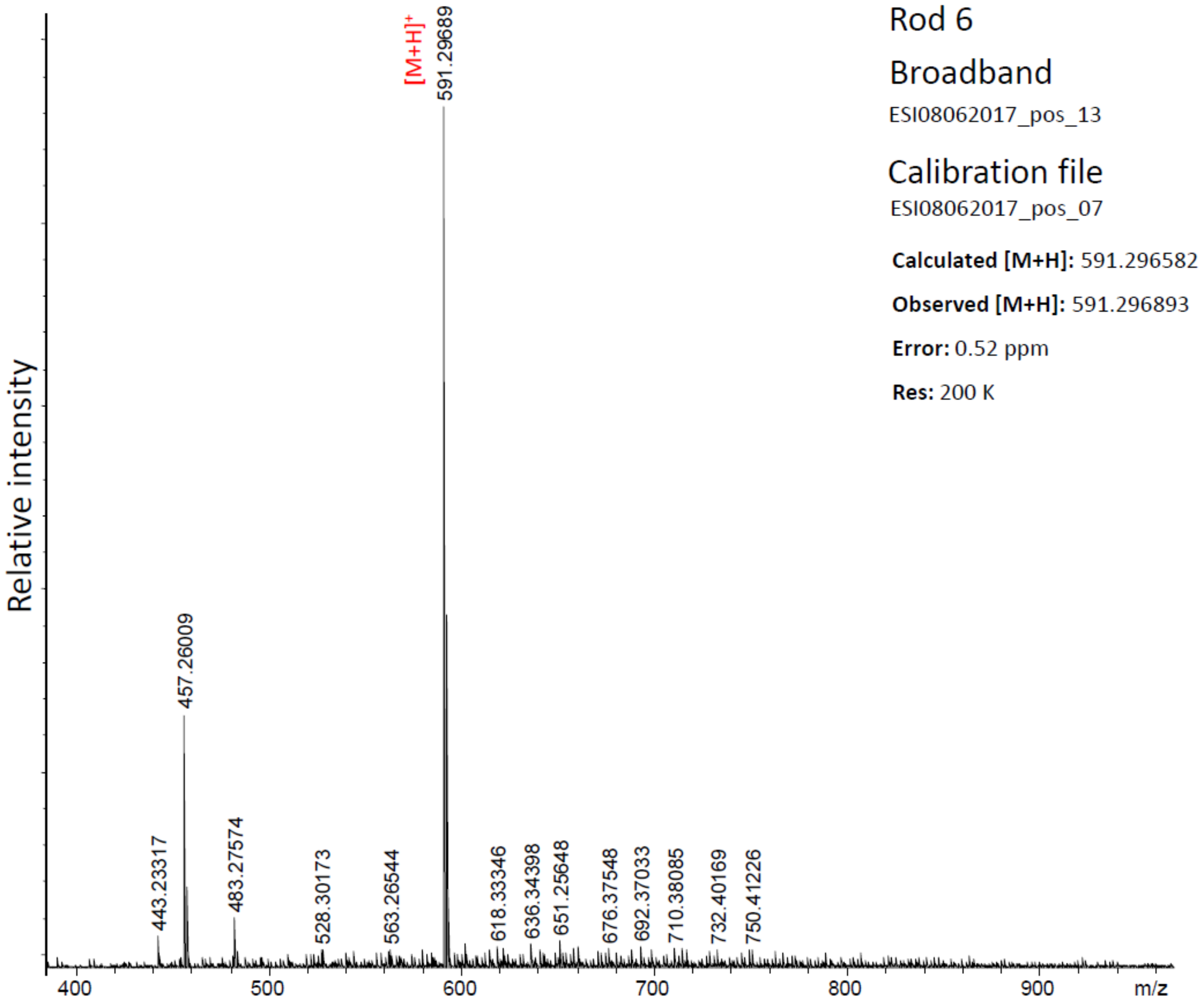

Fig. S5: HRMS (ESI) spectrum of compound **1d**

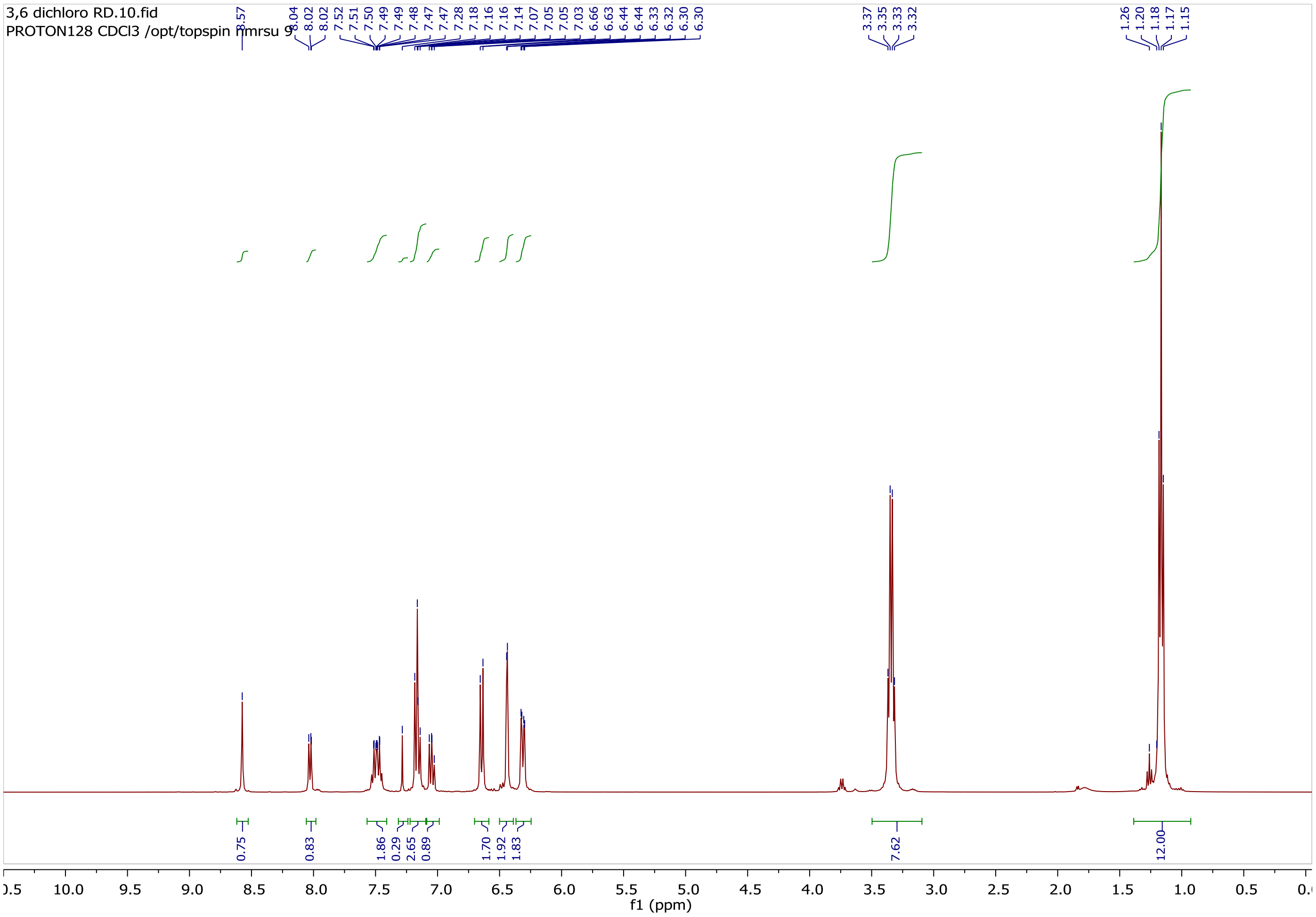

Fig S6: ^{1}H-NMR spectra for compound **1f** ($CDCl_3$, 400MHz)

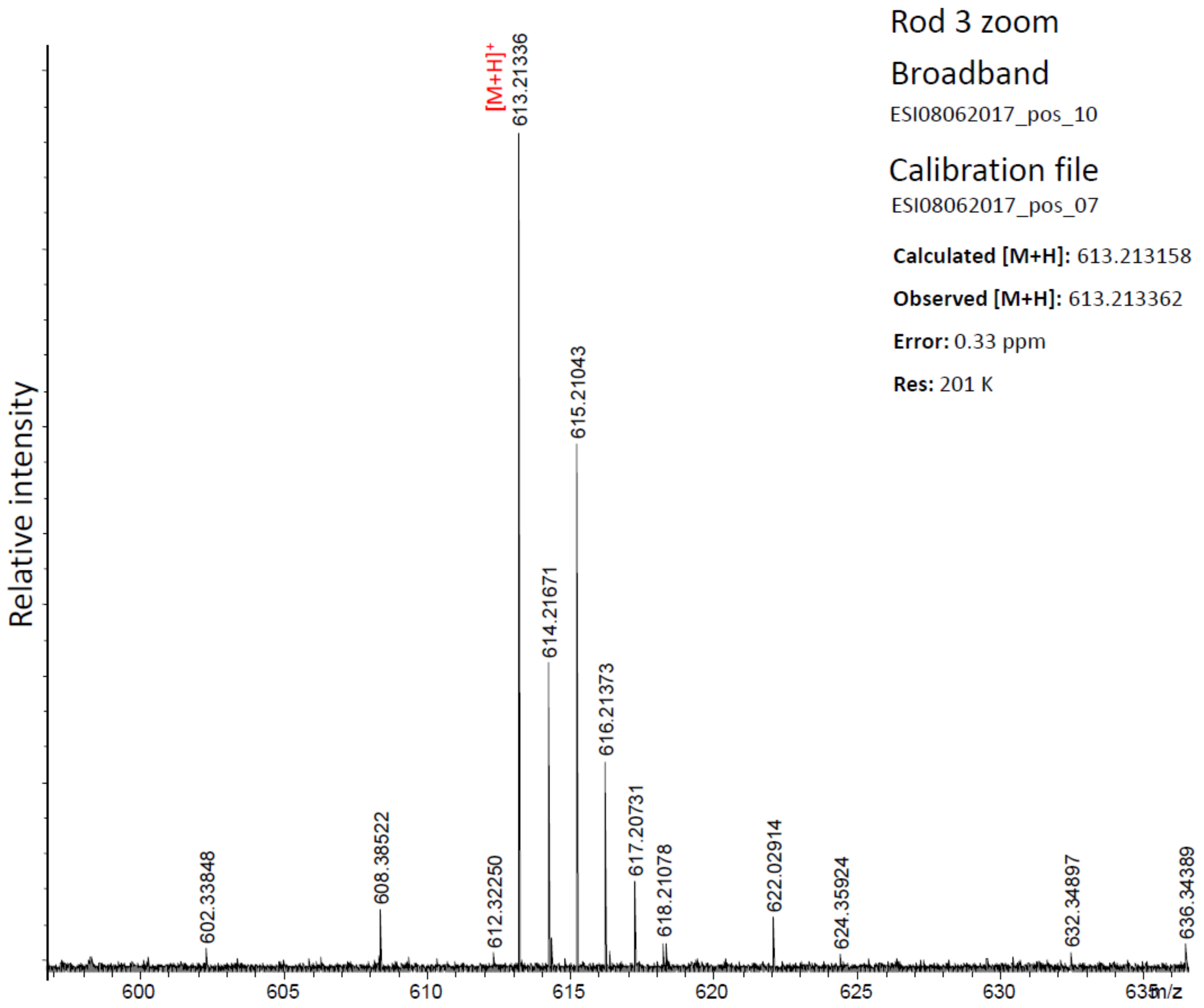

Fig. S7: HRMS (ESI) spectrum of compound **1f**

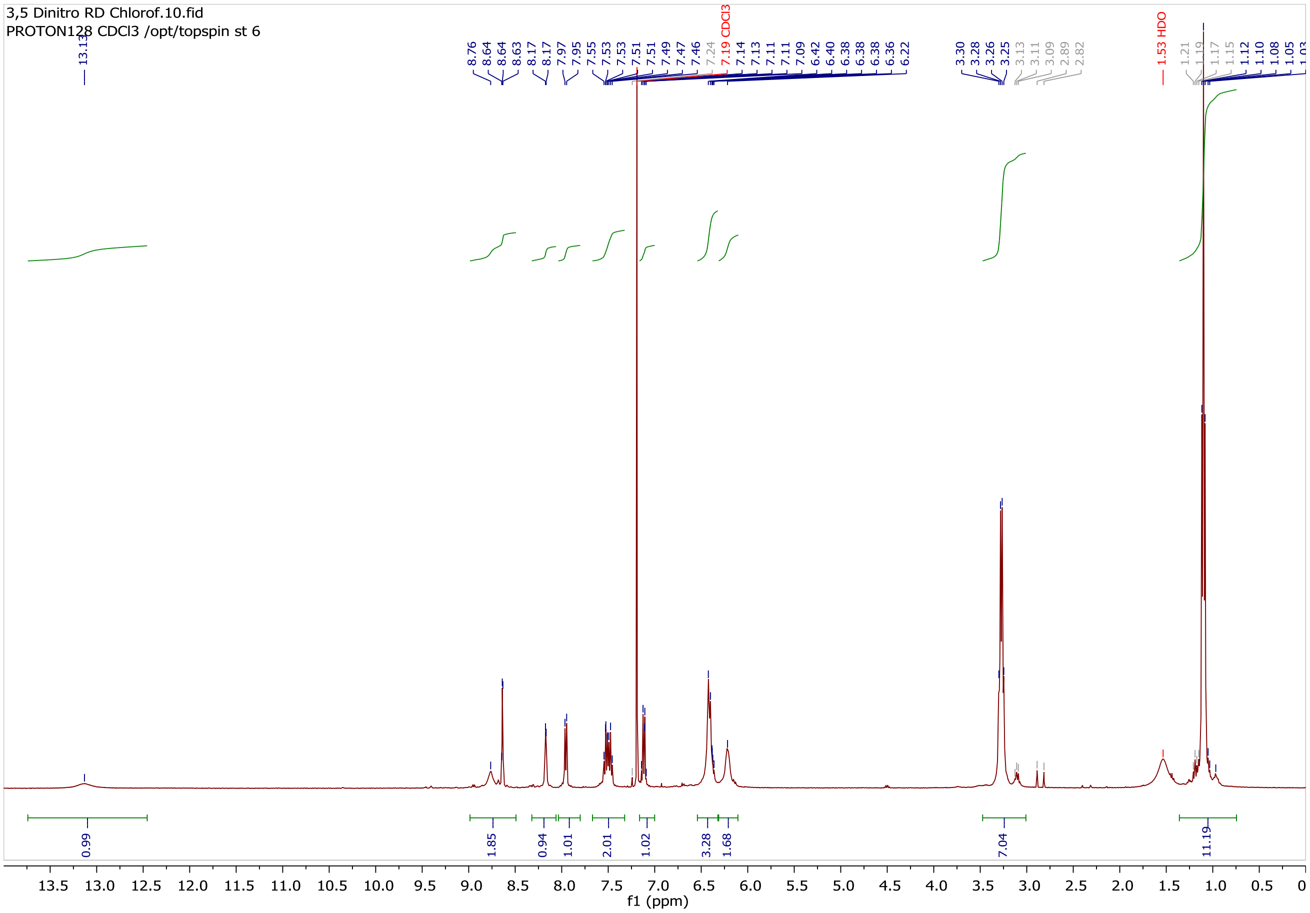

Fig S8: ^{1}H-NMR spectra for compound **1g** ($CDCl_3$, 400MHz)

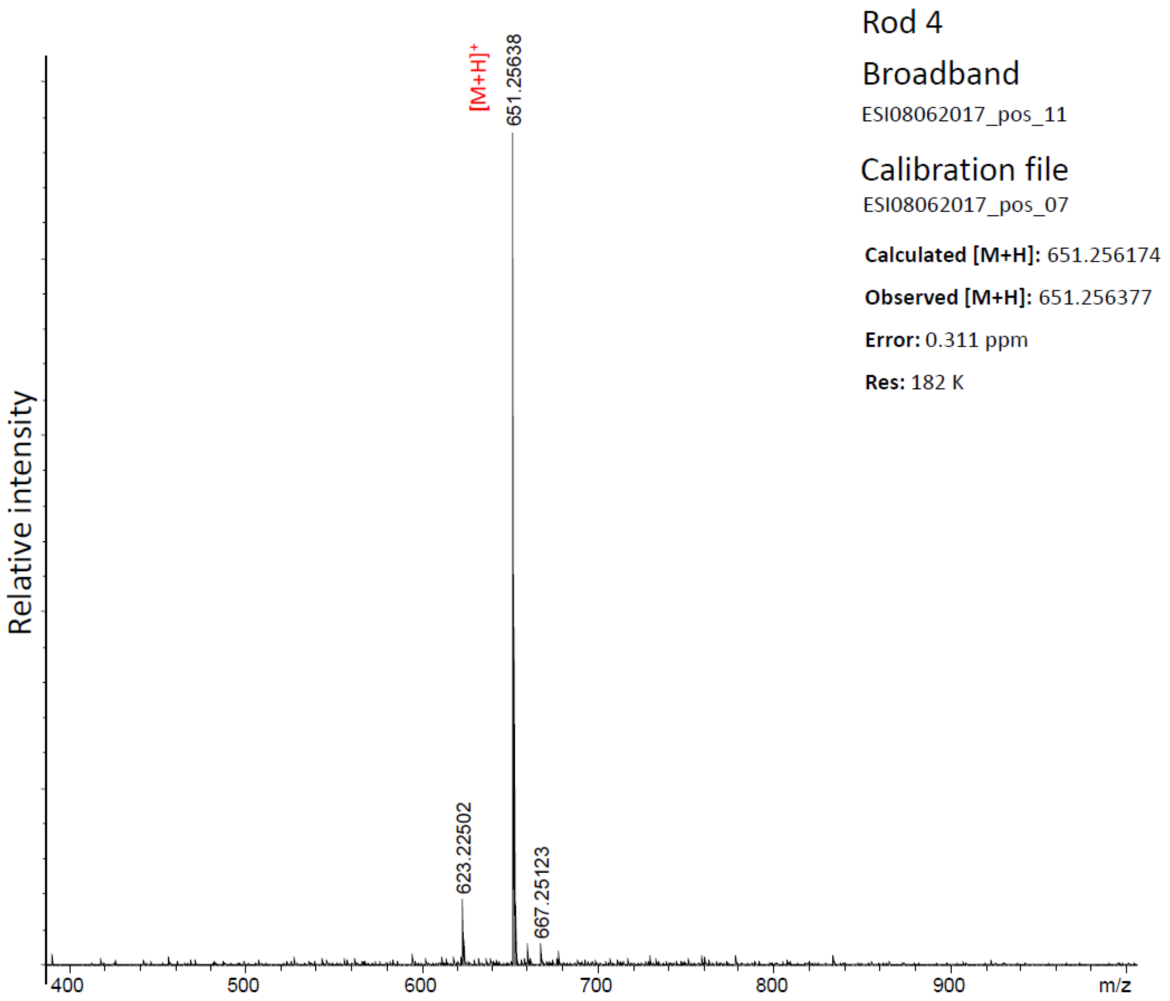

Fig. S9: HRMS (ESI) spectrum of compound **1g**

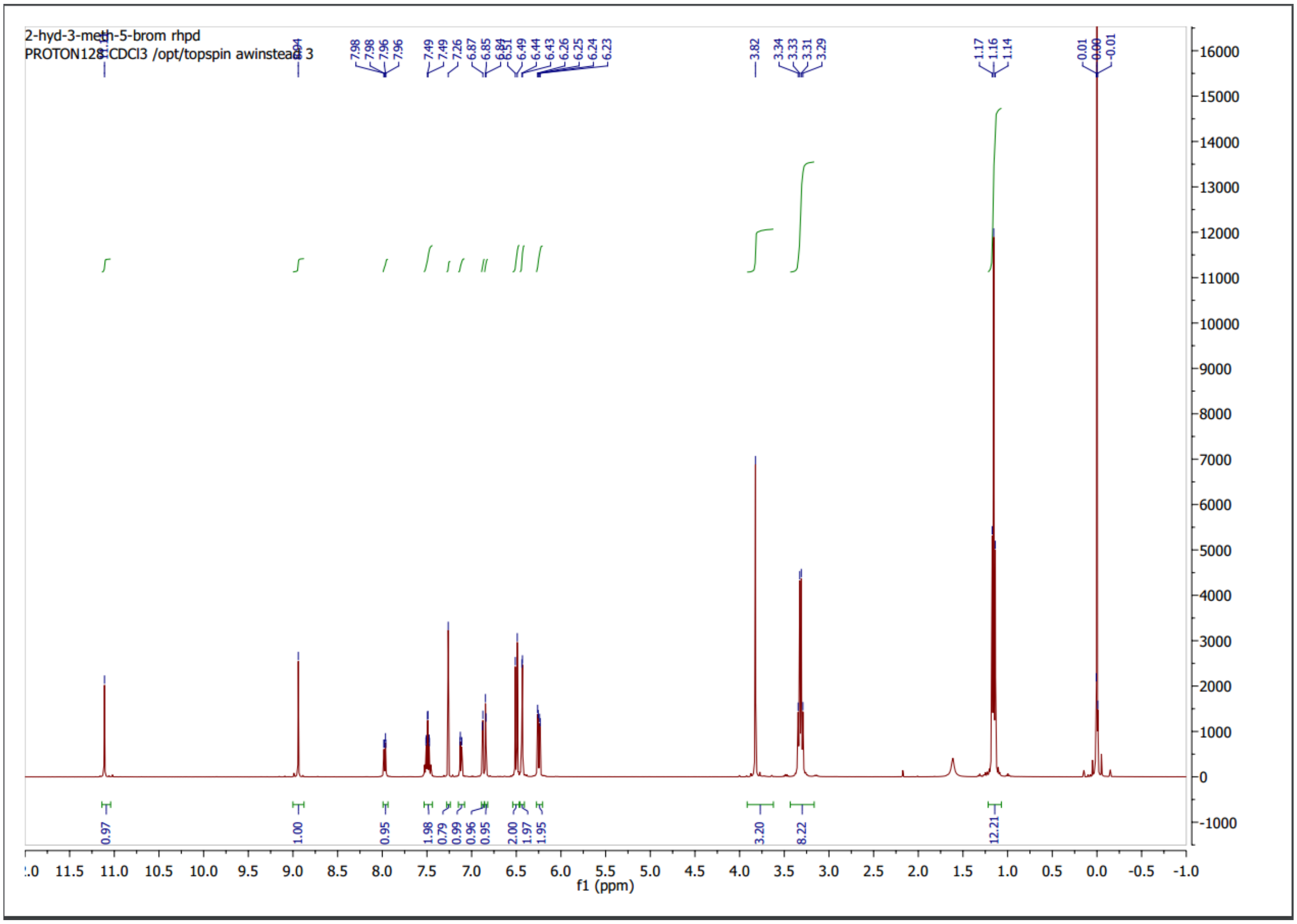

Fig. S10: ^{1}H-NMR spectrum of compound **1h** ($CDCl_3$, 400MHz)

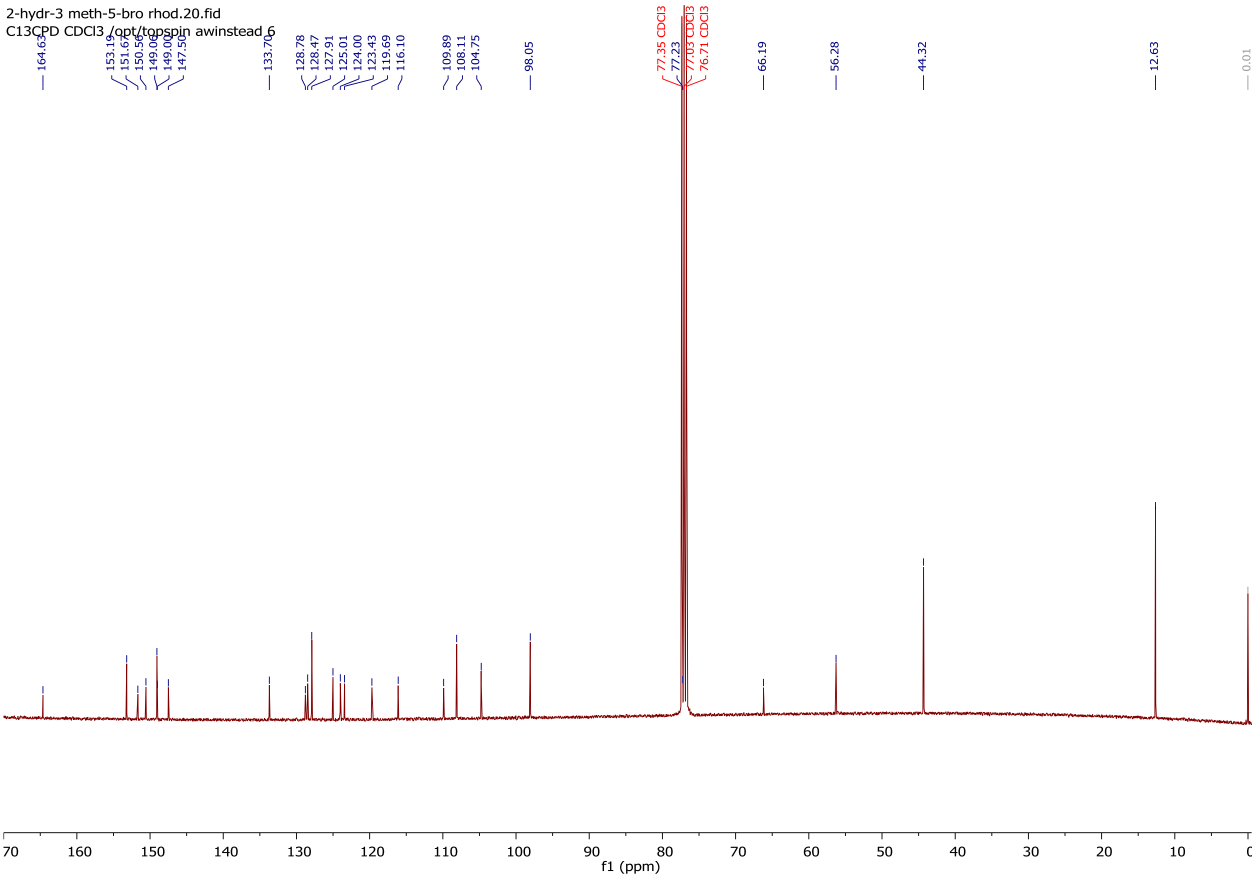

Fig. S11: ^{13}C-NMR spectrum of compound **1h** ($CDCl_3$, 400MHz)

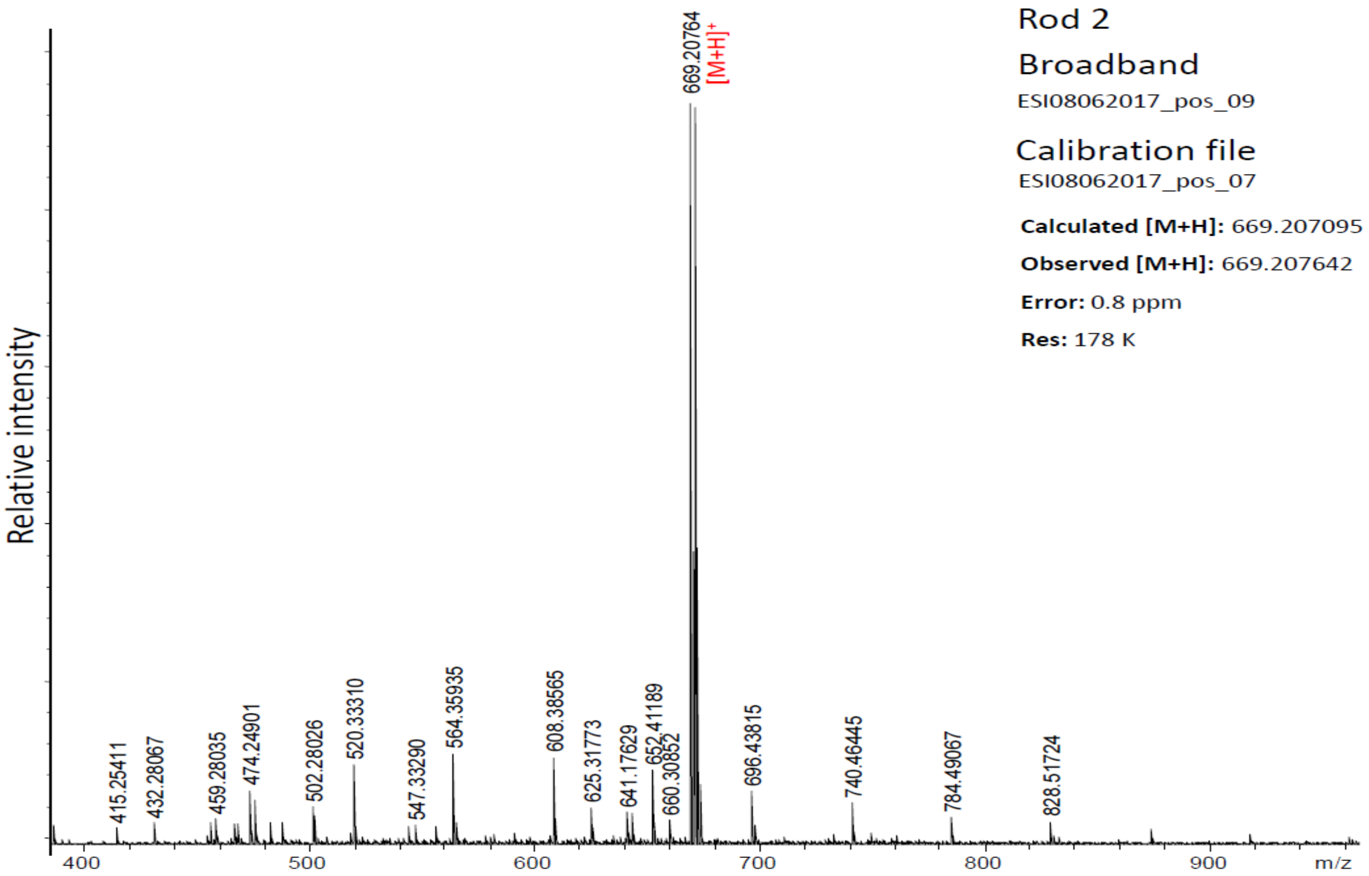

Fig. S12: HRMS (ESI) spectrum of compound **1h**

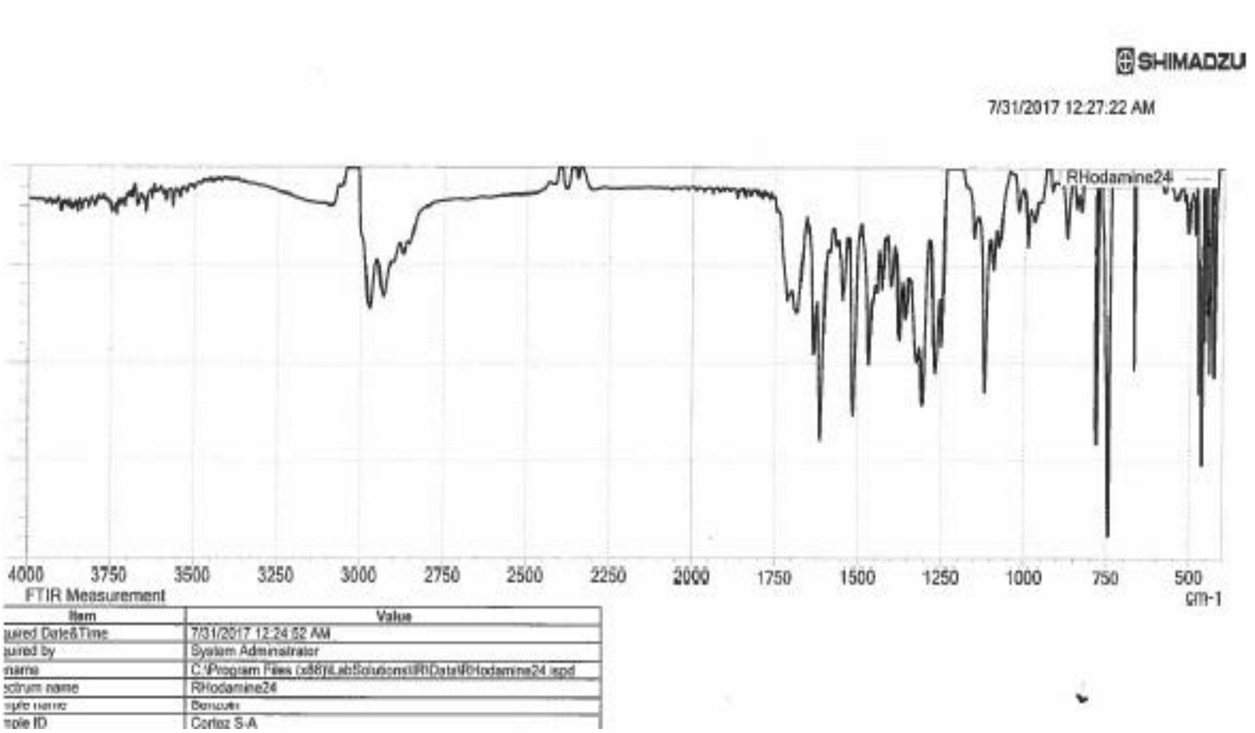

Fig. S13: FTIR spectrum of compound **1h**

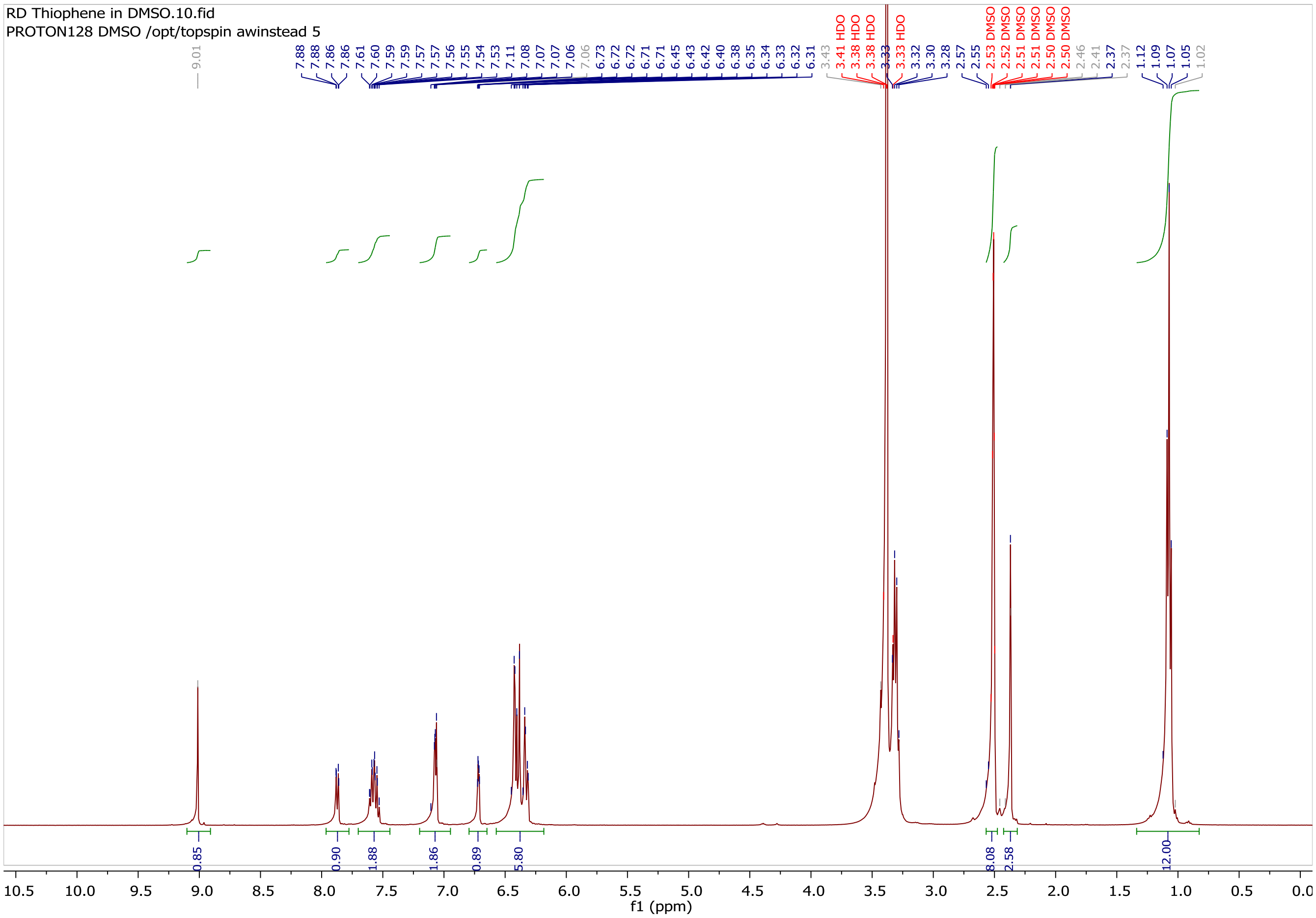

Fig. S14: ^{1}H-NMR spectrum of compound **2** (DMSO-d_{63}, 400MHz)

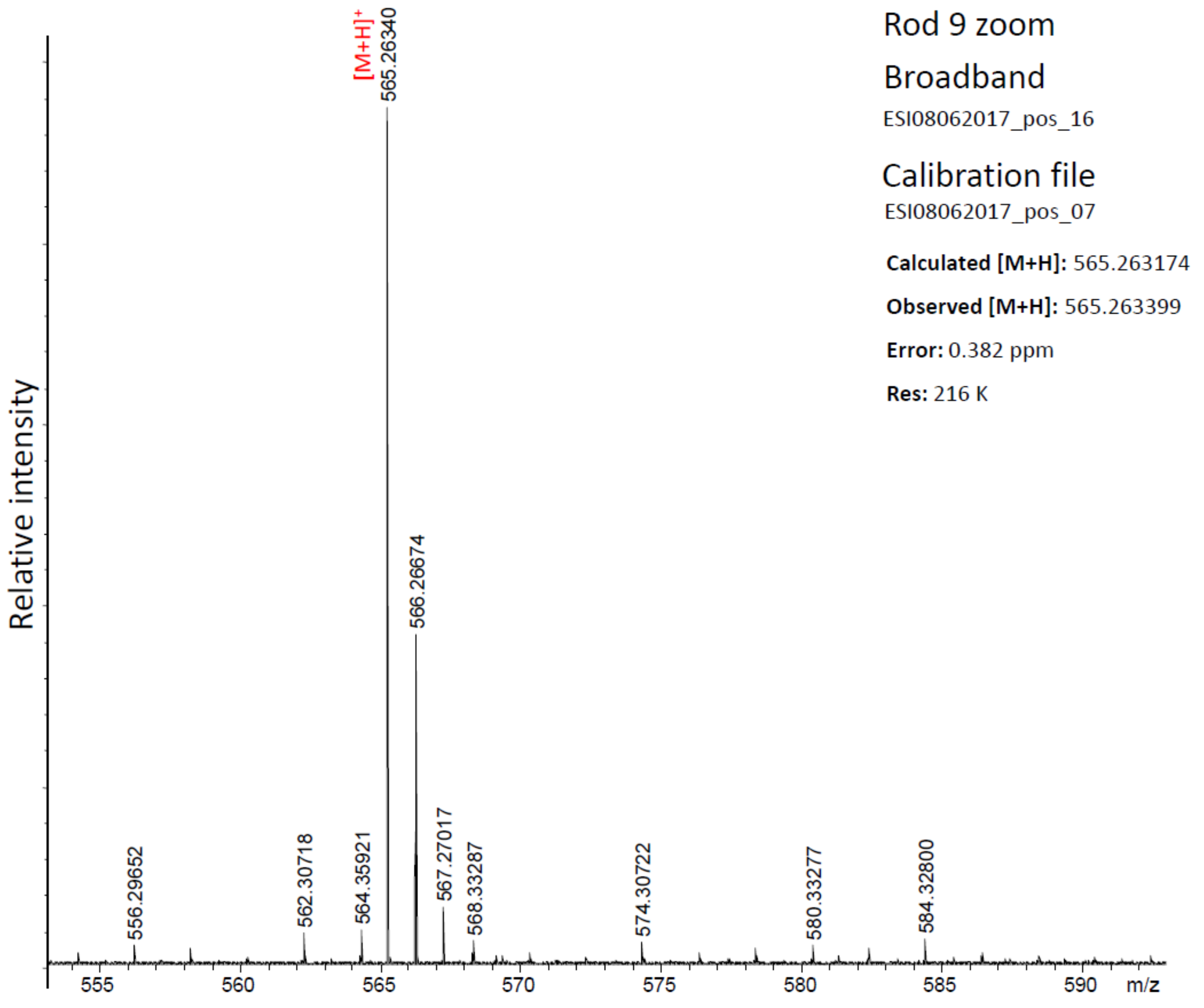

Fig. S15: HRMS (ESI) spectrum of compound **2**

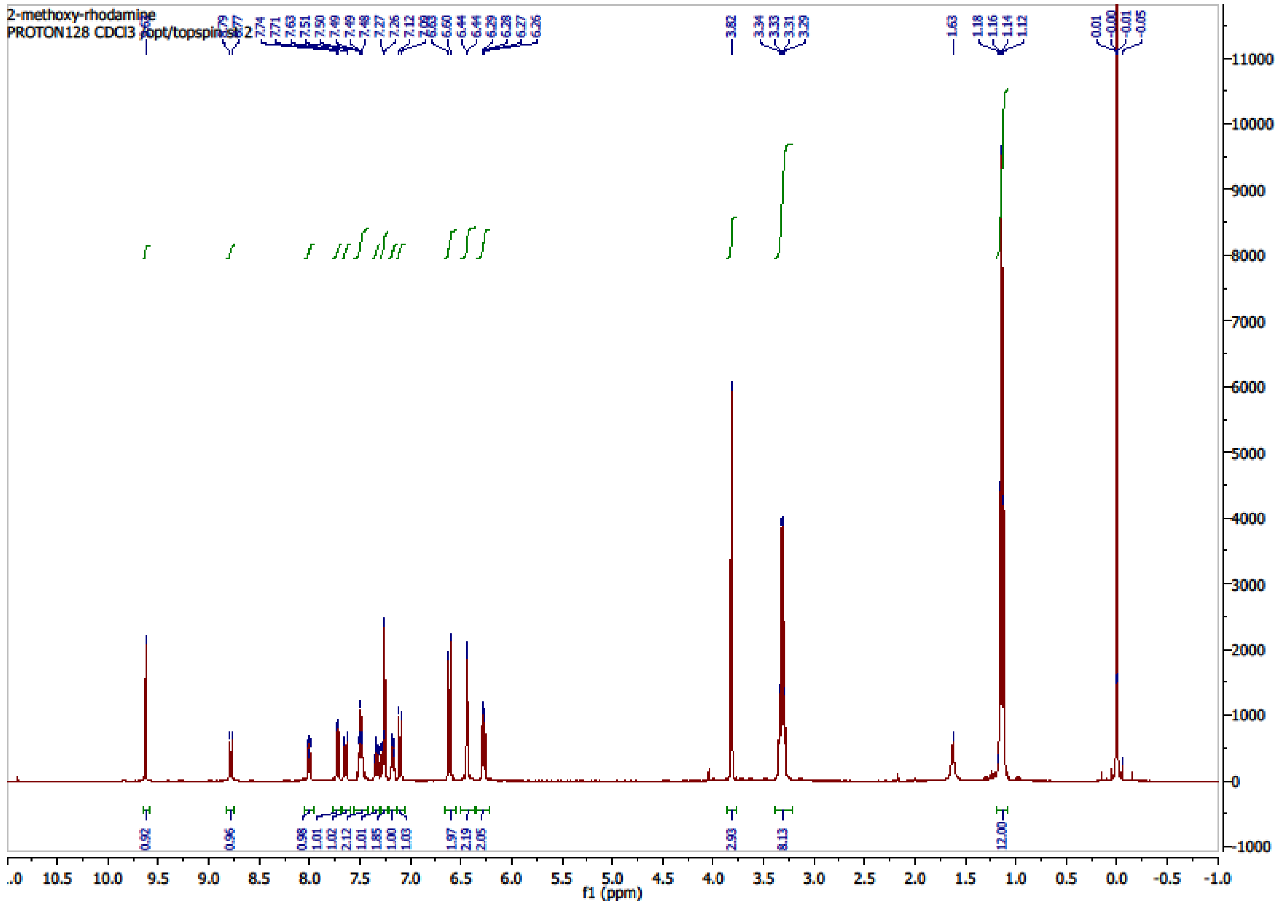

Fig. S16: ^{1}H-NMR spectrum of compound **4** ($CDCl_3$, 400MHz)

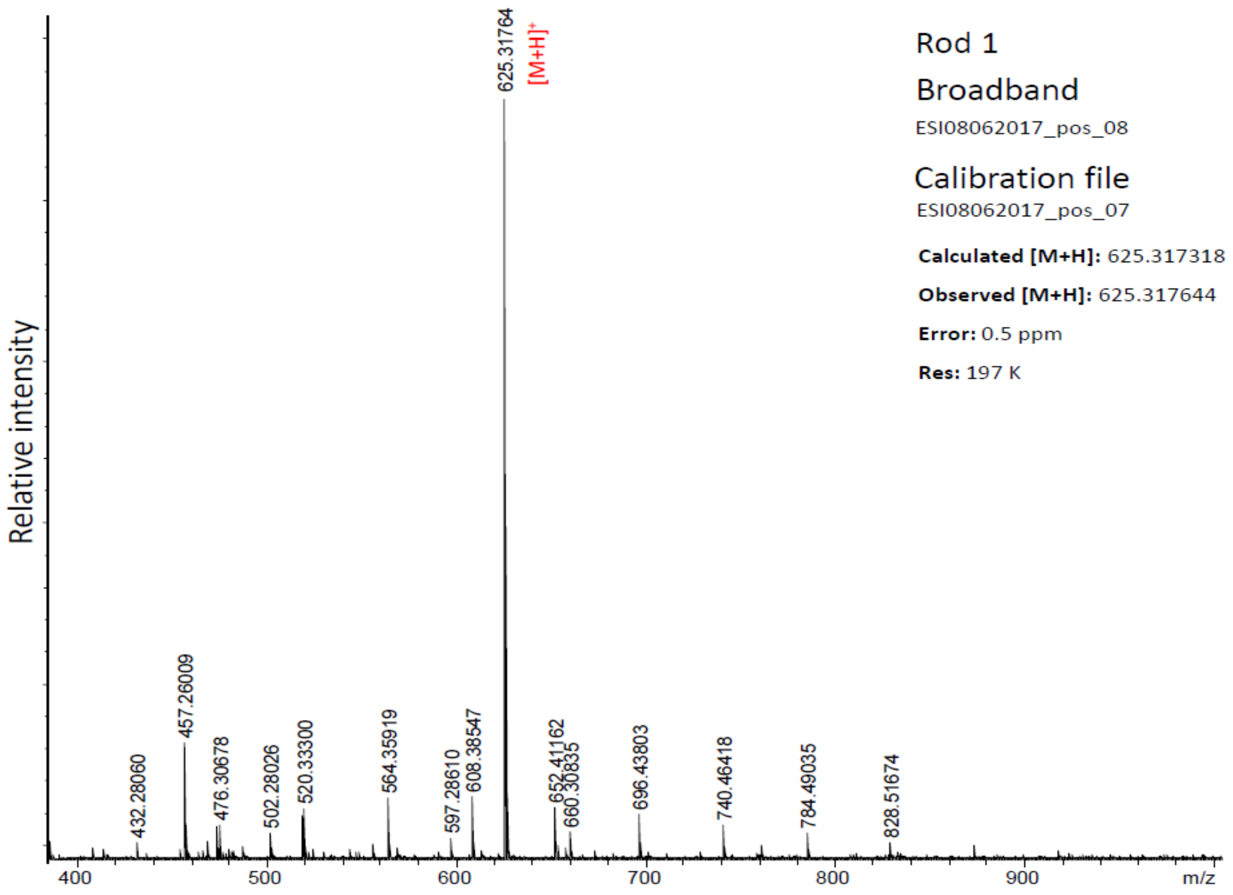

Fig. S17: HRMS (ESI) spectrum of compound **4**

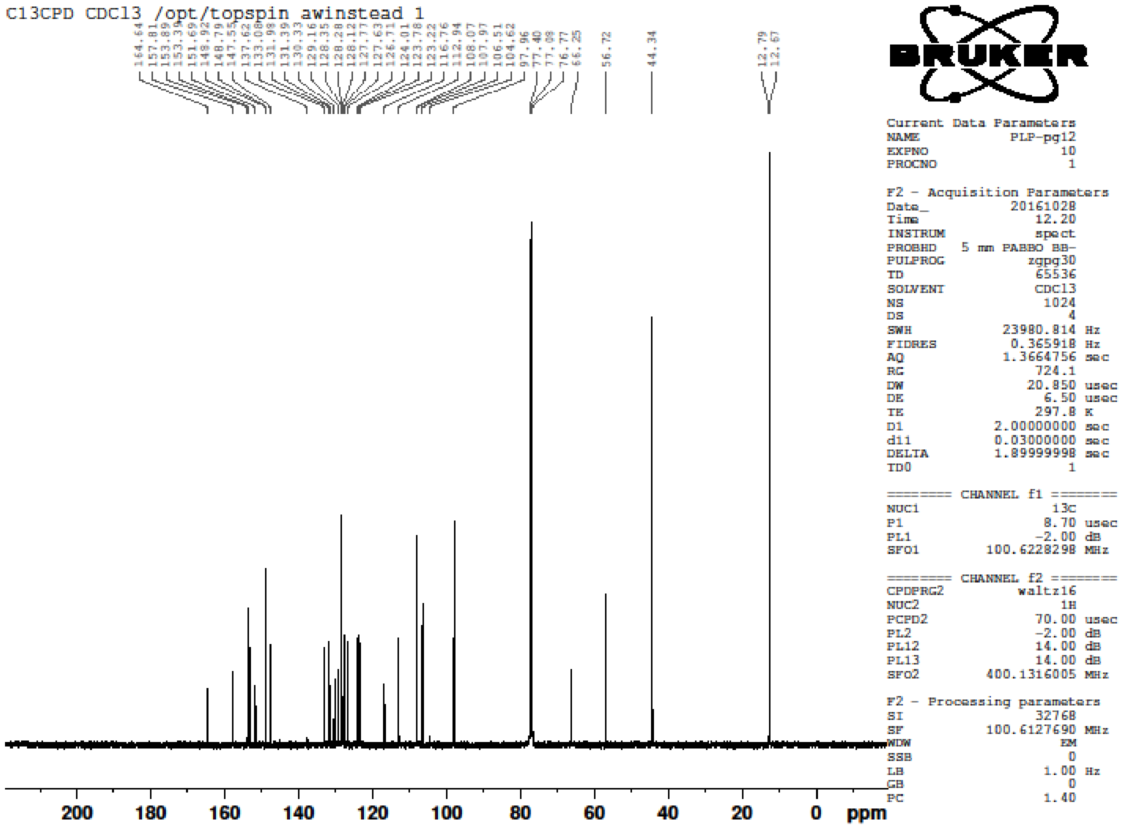

Fig. S18: ^{13}C-NMR spectrum of compound **4** ($CDCl_3$, 400MHz)

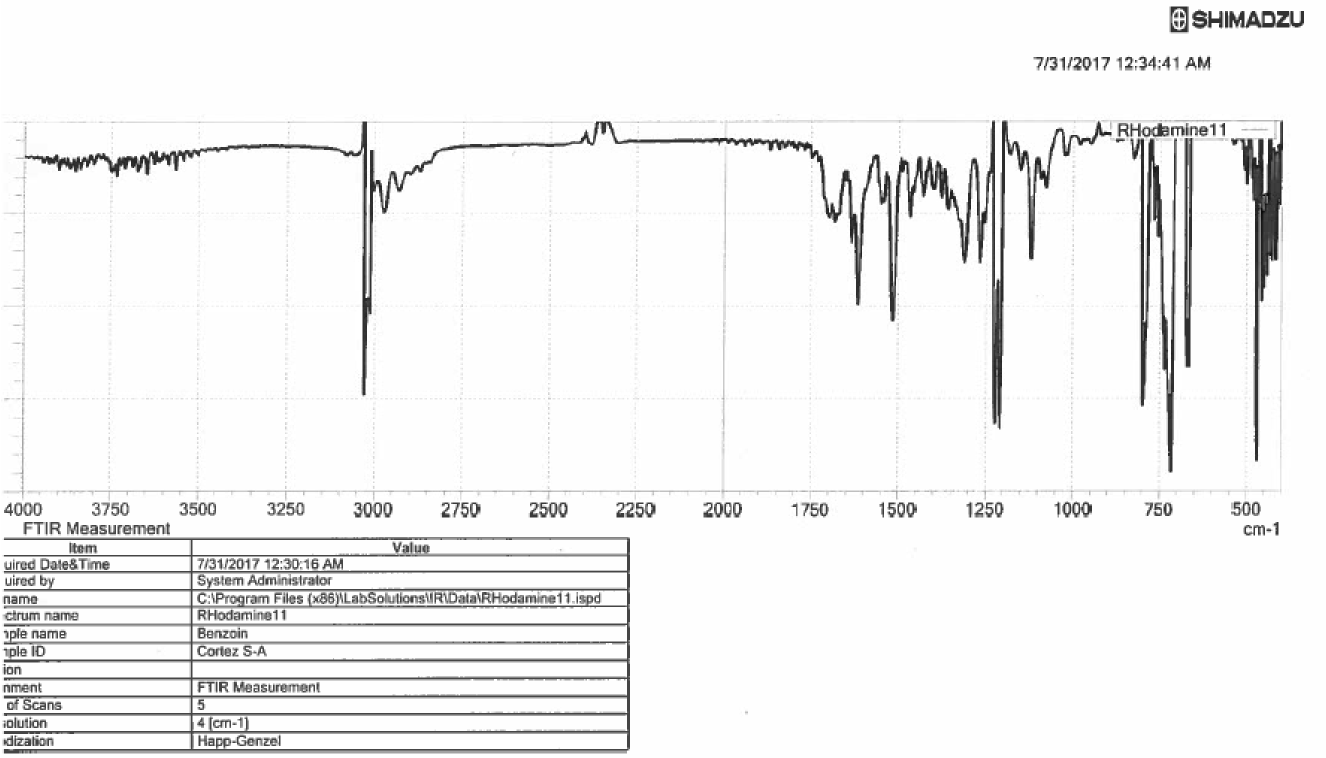

FTIR Measurement

Item	Value
uired Date&Time	7/31/2017 12:30:16 AM
uired by	System Administrator
name	C:\Program Files (x86)\LabSolutions\IR\Data\RHodamine11.ispd
ctrum name	RHodamine11
nple name	Benzoin
nple ID	Cortez S-A
ion	
nment	FTIR Measurement
of Scans	5
solution	4 [cm-1]
dization	Happ-Genzel

Fig. S19: FTIR spectrum of compound **4**

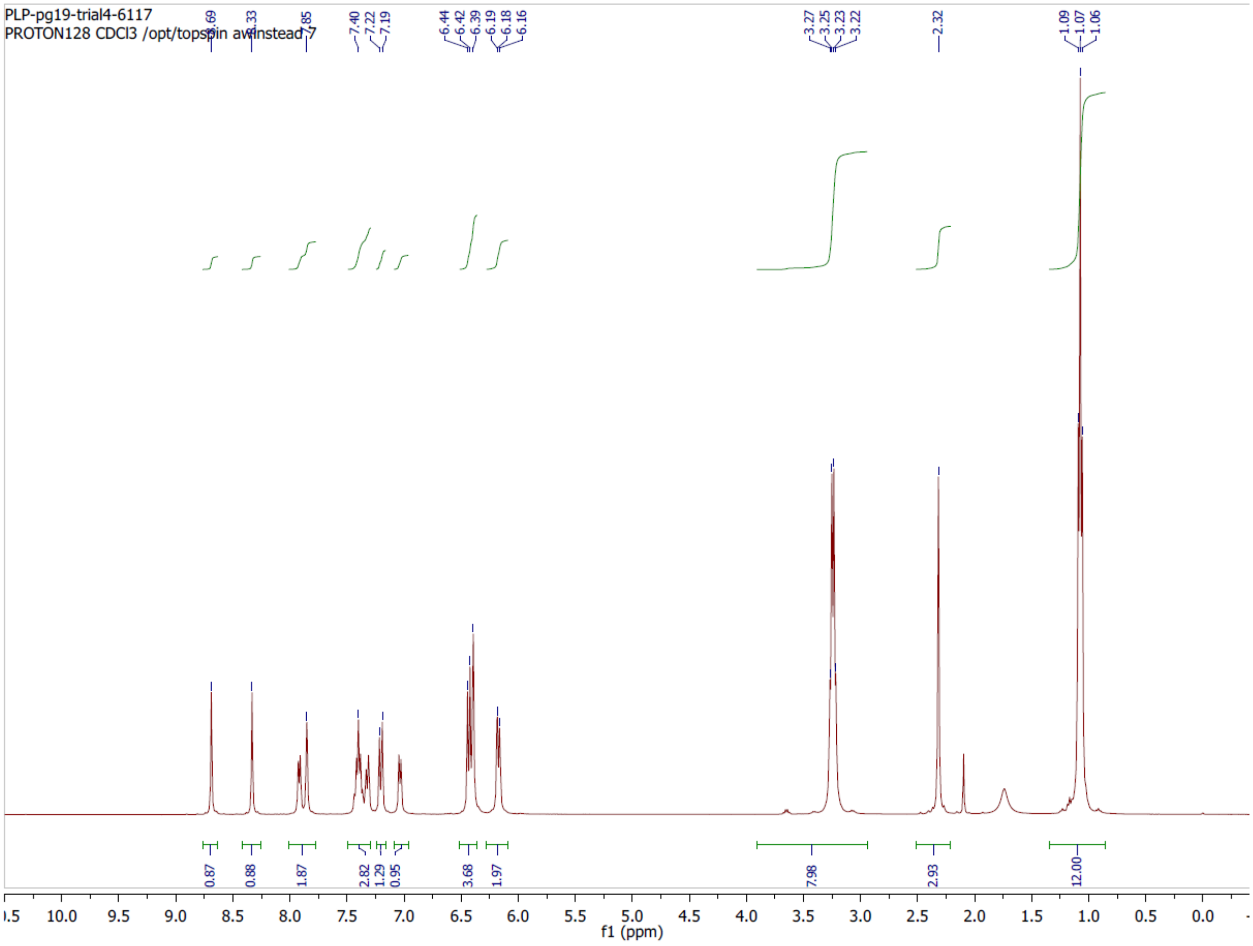

Fig. S20: ^{1}H-NMR spectrum of compound **5** ($CDCl_3$, 400MHz)

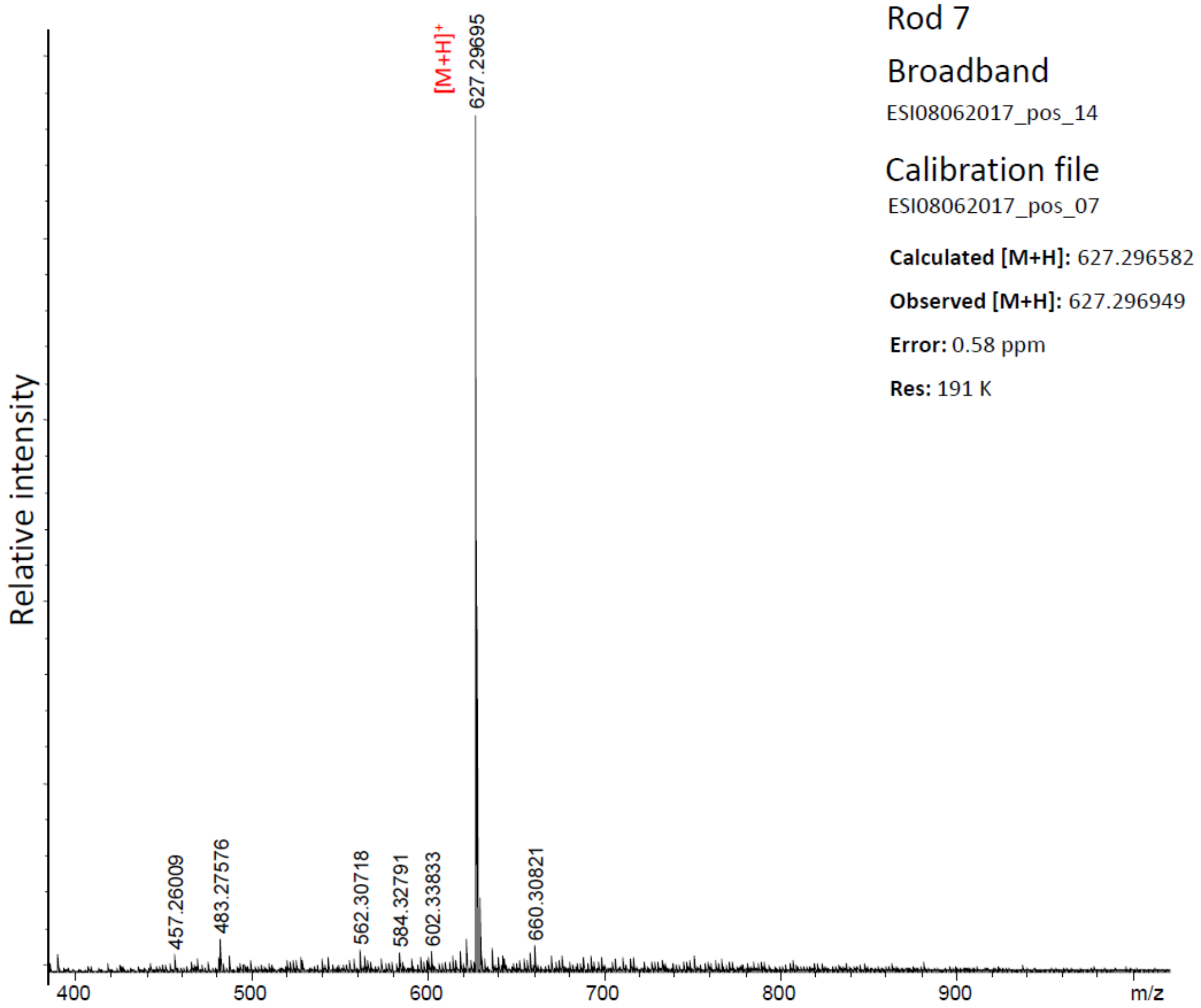

Fig. S21: HRMS (ESI) spectrum of compound **5**
